# Sulfated chitosan directs the recovery of ischemic stroke by attenuating the inflammatory cascade

**DOI:** 10.7150/thno.111681

**Published:** 2025-04-28

**Authors:** Zehua Gao, Xuanlin Wang, Qiyong Mei, Tong Shen, Jing Wang, Changsheng Liu

**Affiliations:** 1 The State Key Laboratory of Bioreactor Engineering, East China University of Science and Technology, Shanghai 200237, China.; 2 Key Laboratory for Ultrafine Materials of Ministry of Education, East China University of Science and Technology, Shanghai 200237, China.; 3 Engineering Research Center for Biomedical Materials of Ministry of Education, East China University of Science and Technology, Shanghai 200237, China.; 4 Department of Neurosurgery, Changzheng Hospital, Naval Medical University, Shanghai 200003, China.

**Keywords:** sulfated chitosan, inflammation, neutrophil, ischemia stroke

## Abstract

**Background:** Ischemic stroke is considered a fatal ischemic disease with high mortality and morbidity. Acute ischemic stroke is a cascade of inflammatory reactions, which not only causes vascular degeneration but also leads to neurological disorders. During this period, the rapid response of neutrophil-dominated granulocytes releases cytokines and chemokines to affect tissue repair. Thus, effective regulation of neutrophils appears to be the key in treating major organ injuries associated with inflammation.

**Methods:** This study developed a semisynthetic sulfated chitosan (SCS) associated with the functional sulfated groups. The immunoregulatory effects of SCS on neutrophils were tested by Real-Time Quantitative Reverse Transcription (RT-PCR), ELISA and immunofluorescence staining at gene and protein levels *in vitro*. Flow cytometry, WB and PCR were used to study the effect of neutrophils on macrophages, indicating the regulation of the inflammatory cascade by SCS. Acute ischemic stroke model was established to verify the effectiveness and the regulation of inflammatory cascade of SCS. Finally, the lower limb ischemia model was used to verify the universality of SCS in the treatment of ischemic diseases, especially with regard to acute inflammatory-related major organ damage.

**Results:** SCS can not only promote neutrophil apoptosis, but also enable neutrophils to produce vascular-related subsets to regulate immunity and promote angiogenesis. Neutrophil stimulated by SCS mediated macrophage polarization via IL-10-induced Stat3 signaling pathway to weaken the inflammatory cascade. In animal models of ischemic hind limb and ischemic stroke, SCS had demonstrated its ability to shorten the acute inflammatory period, as indicated by neutrophil, and accelerate the subsequent repair period characterized by the presence of M_2_ macrophages. Additionally, SCS effectively inhibits the expression of MMP-9 to provide a favorable environment for rapid extracellular matrix reconstruction. Encouragingly, treatment with SCS had been shown to reduce the expansion of the infarct volume by approximately 20% in our experiments.

**Conclusion:** This study underscores the effect of SCS in regulating the heterogeneity of neutrophils in order to suppress the initiation of inflammation to treat ischemic stroke. Crucially, our approach relies on non-exogenous growth factors and cells, highlighting its remarkable potential for clinical translatability in the treatment of major organ injuries.

## 1. Introduction

Ischemic stroke is a significant global cause of mortality and long-term disability 1,2. The acute phase of this condition is characterized by blood vessel occlusion, which further exacerbates neuronal necrosis and brain dysfunction due to severe inflammation. Currently, the standard treatment for ischemic stroke in clinical settings involves the use of thrombolytic drugs or thrombectomy procedures 3. These interventions aim to achieve reperfusion and rescue the ischemic penumbra 4. However, these strategies are limited by a narrow therapeutic window and hemorrhagic complications 5. Furthermore, reperfusion itself can lead to secondary injuries, inducing overproduction of inflammation and reactive oxygen species (ROS) in the cerebrovascular system and neural networks 6,7. Attempts have been made to supplement the standard treatment with exogenous growth factors or neuroprotective agents to promote angiogenesis and neuroprotection. However, the clinical application of these agents is hindered by challenges such as low bioavailability, short half-life, and limited ability to penetrate the blood-brain barrier (BBB) 8. In order to address these limitations, some biomaterials with cells or factors have been developed 7,9. However, the potential biosafety risks associated with these biomaterials raise concerns. Therefore, there is an urgent need for novel treatment strategies that can effectively promote rapid vascularization and regulate inflammation in ischemic stroke cases.

Immunomodulatory mechanisms play a crucial role in the pathophysiology of ischemic/reperfusion (I/R) injuries during stroke 10. Reperfusion following ischemia triggers a cascade of inflammatory reactions 11, where the swift response of neutrophils-dominated granulocytes in the acute inflammatory phase leads to the release of cytokines and chemokines 12. Consequently, infiltrating macrophages and monocytes from the peripheral blood activate brain resident immune cells, namely microglia and astrocytes, exacerbating the hyperinflammatory conditions in the infarcted area 13,14. In light of this, the spatio-temporal regulation of immune cells emerges as an ideal therapeutic strategy for cerebrovascular injury 15. During the acute phase of stroke, neutrophils adhere to the endothelial lining of brain blood vessels and subsequently enter the brain parenchyma through the blood-brain barrier, initiating immune responses and regulatory processes. It is important to note that the heterogeneity of neutrophils can yield varying effects on stroke recovery 16. Accordingly, modulating the behavioral changes of neutrophils presents an opportunity to mediate the onset of the inflammatory cascade, restrain neuroinflammation, improve the microenvironment, and ultimately alleviate ischemic brain injury. Consequently, immunotherapy holds promising prospects in the treatment of stroke.

Sulfated glycosaminoglycan (GAG) is a commonly found component in the extracellular matrix of mammals 17. With its highly sulfated and anionic properties, GAG can interact with various growth factors, cytokines, and chemokines, forming a chemokine gradient. This gradient plays a crucial role in controlling cell activation and migration 18-20. Matrix metalloproteinase 9 (MMP 9), a major member of the matrix metalloenzyme family, can degrade extracellular matrix to release these factors. However, excessive expression of MMP-9 can accelerate the degradation of the extracellular matrix, which is detrimental to stroke recovery 21. Conversely, appropriate levels of MMP-9 have been found to be beneficial for vascular anastomosis 12. By precisely manipulating the biophysical and biochemical cues of GAG, it is possible to induce endogenous cell-mediated tissue regeneration 22. Through this mechanism, GAG-based engineering materials have the potential to modulate the extracellular matrix and effectively alleviate inflammation. Sulfated chitosan (SCS), a glycosaminoglycan-like polysaccharide, has previously been investigated for its role in regulating inflammation and angiogenesis, such as macrophage polarization 23,24. Building upon these previous findings, we hypothesize that targeting the initiation of inflammation offers a favorable approach to modulate the immune response. SCS may potentially participate in immunomodulation and influence the inflammatory cascade.

**Scheme 1:**
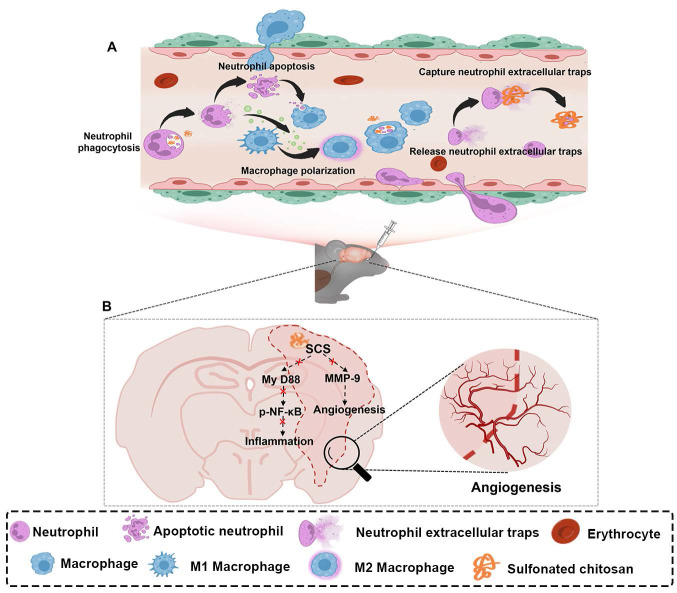
Illustration of synthesized sulfonated chitosan (SCS) for the treatment of ischemic stroke. (A) SCS mediates the regulation of inflammatory cascade by neutrophils and macrophages. (B) Potential molecular mechanism of SCS in the treatment of ischemic stroke.

In this study, we proposed effective immunotherapy strategies to treat stroke. Utilizing bioactive materials modulate the pathological microenvironment by attenuating the initiation of the inflammatory cascade and promoting angiogenesis (scheme 1). SCS, a glycosaminoglycan-like polysaccharide, can enhance the apoptosis of neutrophils. Furthermore, SCS-mediated neutrophil recruitment results in a large number of macrophages polarizing into the M_2_ phenotype. This suggests that SCS can effectively shorten the acute inflammatory period and accelerate the repair process. Additionally, SCS facilitates the release of IL-10 from neutrophils, which further promotes the polarization of macrophages towards M_2_ in ischemic stroke. Therefore, the utilization of bioactive materials to attenuate the onset of the inflammatory cascade and promote angiogenesis, without relying on exogenous growth factors and cells, presents a promising therapeutic strategy for the treatment of ischemic stroke.

## 2. Materials and Methods

### 2.1 Materials

Chitosan, with a molecular weight (Mw) of 3 × 10^5^ Da and deacetylation degree of 92%, was obtained from Shenzhen Zhongfayuan Biological Technology (Shenzhen, China). Chlorosulfonic acid, N, N-dimethylformamide, formamide were purchased from Sinopharm Chemical Reagent Co. (Shanghai, China). Carboxylated chitosan and hydroxypropyltrimethyl ammonium chloride chitosan were purchased from Yuanye Biotech. Co., Ltd. (Shanghai, China). Gelatin from porcine skin was purchased from Sigma (St. Louis, MO, United States). PrimeScript RT reagent kit and SYBR Premix Ex TaqTM were purchased from Takara Biotechnology Co., Ltd. (Dalian, China). Sulfated chitosan (SCS), methacrylated gelatin (GelMA), and initiator were synthesized according to previously described methods 12,25. All cell-culture-related reagents were purchased from Gibco (Grand Island, NY, United States).

### 2.2 Synthesis of Sulfated chitosan (SCS)

SCS was synthesized according to previously described methods 25. All reactions take place in an anhydrous and oxygen-free environment. In brief, the synthesis of SCS is divided into two steps. The first step is the synthesis of sulfonation reagent. The sulfating reagent is obtained by adding 5 mL chlorosulfonic acid to 50 mL N, N-dimethylformamide in an ice bath environment, and then stirring for 30 min. The second is the sulfonation of chitosan, in which 2.5 g of chitosan is dissolved with 50 mL of formamide and 2 mL of formic acid. The sulfating reagent was added to the chitosan solution drop by drop under mechanical agitation, and the reaction is heated to 50 ℃ for 2 h. After the end of the reaction, the product was alternately precipitated and dissolved with ethanol and water, and repeatedly purified twice. The supernatant was collected after centrifugation, the pH was adjusted to 7.2-7.4, and dialyzed against water with a 14 000 Da Mw cut-off dialysis membrane for three days. Finally, the sulfonated chitosan was obtained after lyophilization.

### 2.3 The biosafety of SCS

2 × 10^3^ RAW 264.7 and HUVECs were seeded in 96-well plate. After adherent growth, 2, 4, 8, or 16 mg/mL SCS were added. After cultivation for 1 day, 3 days, and 5 days, cell viability was performed at days 1, 3 and 5 using CCK-8 kit.

### 2.4 MCAO ischemia-reperfusion models

Male C57BL/6 mice were provided by jsj-lab Co. Ltd. (Shanghai, China). All surgical procedures were approved by Institutional Animal Care and Use Committee of East China University of Science and Technology. The transient focal ischemia-reperfusion model was established in mice by blocking the right middle cerebral artery for 60 min using a siliconized filament. Reperfusion was achieved by carefully removing the filament and ligating the wound from the right middle cerebral artery. Upon awakening, the mice were randomly assigned to two groups. One group received daily administration of PBS, while the other group received SCS, both given once a day for three consecutive days through orbital continuous delivery.

### 2.5 Preparation of neutrophil conditional medium-coated GelMA hydrogel

Murine neutrophils were harvested from femurs as previously described 12. Primary neutrophils at a concentration of 2 × 10^7^ cells/mL were cultured overnight, and non-adherent cells were removed in 6-well plates. Subsequently, the neutrophils were subjected to stimulation with 1000 ng/mL lipopolysaccharide (LPS) for 12 h, washed with PBS three times, and then treated with 4 μg/mL SCS (defined as L+S group). As for SCS group, purified neutrophils were directly treated with 4 μg/mL SCS. After SCS stimulation for 12 h, the medium was collected.

GelMA was synthesized according to previously described methods 12. GelMA hydrogel scaffolds coated with conditioned medium from neutrophils were fabricated. Briefly, a solution containing 10% (w/v) GelMA and 0.1% (w/v) initiator in PBS was prepared and subjected to ultraviolet light crosslinking to form hydrogel. Meanwhile, the L+S group conditioned medium was freeze-dried, 50 μL DMEM was dissolved and then dripped onto the freeze-dried GelMA hydrogel (defined as L+S). The freeze-dried GelMA hydrogel was dripped with 50 μL DMEM (defined as DMEM) or PBS (defined as PBS) as control group. Finally, they were freeze-dried again for lower limb ischemia model.

### 2.6 Mouse ischemic hind limb model

After the administration of isoflurane anesthesia, the hair was removed, and an incision was made on the right femoral skin. The nerves and veins are separated from the femoral artery under a stereomicroscope (RWD). With the ligation of two knots, the femoral artery was then transected at its midpoint. Following this procedure, pre-coated GelMA hydrogel scaffolds were implanted into the muscle beneath the vascular wound. The progression of vascular perfusion within the implanted region was evaluated using Laser Speckle Contrast Imaging on both day 7 and day 14.

### 2.7 Histology and immunofluorescence

The hydrogel scaffolds underwent a series of steps for processing and analysis. First, they were fixed in a neutral paraformaldehyde buffer for 4 h. Then, they were immersed in a solution consisting of 20% sucrose and 2% PVP overnight. Next, the scaffolds were embedded in OCT (Leica, Germany) and sectioned at a thickness of 10 μm. For histological analysis, the sections were stained with H&E. On the other hand, for immunofluorescence analysis, the sections were permeabilized and then blocked with 5% goat serum at room temperature for 1 h. Following the blocking step, the sections were incubated with various antibodies overnight at 4 ℃, such as F4/80, CD31, Ly 6G, H3Cit, and GLUT. Subsequently, appropriate secondary antibodies were added and allowed to incubate. Finally, the nuclei were stained with anti-fade DAPI.

### 2.8 Flow cytometry

Following euthanasia, the implantation and brain tissues were carefully removed and cut and ground in PBS solution. Subsequently, cell suspensions were obtained by passing the tissues through a 40-μm strainer, followed by centrifugation and washing using cell staining buffer. The resulting single-cell suspensions were incubated with different specific antibodies at a temperature of 4 °C for 30 min. Following a final wash step, the cells were analyzed using a Cytoflex LX flow cytometer (Beckman Coulter, United States).

### 2.9 Cell viability, phenotype, and cell apoptosis test

To assess the viability of neutrophils in an *in vitro*, various assays were conducted. The SA-*β*-Gal assay and cell cycle analysis were performed at 0, 24, and 48 h, following the manufacturer's instructions. In order to examine the changes in neutrophil nucleus and phenotype, Wright-Giemsa staining and flow cytometry were utilized. To simulate the inflammatory microenvironment, the isolated neutrophils were stimulated with 1000 ng/mL LPS for 12 h and subsequently washed with PBS three times. To observe the impact on the nucleus, different concentrations of SCS were added for 12 h, followed by fixation with methanol and air-drying. Wright-Giemsa staining was then conducted. For the observation of phenotype, 4 μg/mL SCS or 100 ng/mL TGFβ was added for 12 h, after which flow cytometry analysis was performed as previously described.

Flow cytometry was also utilized in the cell apoptosis assay. Briefly, the isolated neutrophils were cultured with 4 μg/mL SCS for 12 h, after stimulated with 1000 ng/mL LPS for 12 h. Subsequently, a cell apoptosis assay (MX3210, Maokang Biotechnology Co., Ltd, Shanghai) was conducted in accordance with the manufacturer's instructions.

### 2.10 Isolation of peritoneal macrophages

The macrophages were obtained from C57BL/6 male mice through a consistent 3-day injection of 6% Nutrient Broth. Following euthanasia, PBS was intraperitoneally injected and collected. The collected cells were centrifuged and cultured in DMEM supplemented with 10% FBS and 1% penicillin/streptomycin for 4 h. Non-adherent cells were subsequently removed and fresh medium was added. The purity of the cells was assessed using flow cytometry.

### 2.11 RNA analysis by qRT-PCR

qRT-PCR analysis was conducted to investigate the gene expression levels in neutrophils and macrophages. Isolated neutrophils and macrophages were subjected to stimulation with 1000 ng/mL LPS for 12 h, washed with PBS three times. Subsequently, 4 μg/mL SCS was added and incubated for an additional 12 h. The mRNA from neutrophils and macrophages was then extracted, with the concentration determined, normalized, and converted into cDNA through reverse transcription. The expression levels of the target genes were assessed using the CFX96 TouchTM PCR detection system. The Table S1 showed all primer sequences.

### 2.12 Quantification of cytokines

We performed IL4 and IL10 ELISA analysis of neutrophil conditioned medium, plasma, and brain supernatant according to the manufacturers' instructions (Neobioscience). The secretion of angiogenic cytokines was detected using a Proteome Profiler Mouse Angiogenesis Array Kit (R&D Systems).

### 2.13 Neutrophil extracellular traps (NET) staining

Purified neutrophils stimulated with 1000 ng/mL LPS for 12 h, washed with PBS three times, and then 4 μg/mL SCS, chitosan quaternary ammonium salt (HACC), or carboxylation chitosan (CCS) were added for 12 h. After removing medium, the cells were fixed with neutral paraformaldehyde buffer for 15 min. Subsequent staining is the same as immunofluorescence.

### 2.14 The proliferation and migration of HUVECs

2 × 10^3^ HUVECs were seeded in 96-well plate. After adherent growth, the DMEM was replaced with conditioned medium. After cultivation for 1 day, 3 days, and 5 days, cell proliferation assay was performed at days 1, 3 and 5 using CCK-8 kit.

the migratory capacity of HUVECs was investigated by lateral migration assay. Briefly, 1 × 10^4^ HUVECs were seeded and cultured until covered the bottom in 96-well plate.

The scratch wounds were created, removed cell debris, added different conditioned medium containing 2% FBS. At 0, 12, 24, and 48 h after injure, the images were captured, and quantification was evaluated using ImageJ.

### 2.15 *In vitro* polarization of macrophages

The purified peritoneal macrophages were cultured overnight, and the DMEM was replaced with different neutrophil conditioned medium for 1 and 3 days. The polarization of macrophages was determined by flow cytometry.

### 2.16 Analysis of ischemic brain damage

The mice were euthanized at days 1, 3, and 7 after injection to assess the infarct volume. TTC staining was employed for this purpose. Firstly, the brain tissues were rinsed with saline and cut into 2-mm thick slices. These slices were then subjected to TTC staining at 37 °C for 30 min. Following this, the tissue samples were fixed using a neutral paraformaldehyde solution. Additionally, Magnetic Resonance Imaging (MRI) was performed using magnetic resonance imaging dynamic scanning analysis (Chenguang Medical, 7.0T). T2-weighted rapid acquisition with relaxation enhancement (RARE) sequences were used to obtain 13 coronal sections (with a thickness of 0.6 mm) in order to capture the entire brain in the MRI. The infarct volume was measured using a standard Digital Imaging and Communications in Medicine (DICOM) viewing software (RadiAnt DICOM Viewer).

### 2.17 Behavioral tests

To evaluate the recovery of sensorimotor deficits following injection, open field and beam balance tests were performed at 1, 3, and 7 days post-injection. The open field test consisted of a black wooden box with a white floor measuring 40 cm × 40 cm. The floor was divided into 4 × 4 equal squares using painted lines (SA215, Shanghai Qiansu Biotechnology Co., Ltd, China). During a 15-minute observation period, the mice were placed in one corner of the apparatus facing the wall. Their movement within the central area was tracked and recorded. For the beam balance test, custom-made equipment was used. The mice were required to cross a 100 cm long beam with a width of 2.5 cm and positioned 50 cm above the floor. All mice underwent a three-day training period prior to the MCAO. Each mouse was placed at one end of the beam and allowed a maximum of 120 s to cross. The time taken to cross the beam and the number of foot slips were analyzed. The performance of the mice was then scored on a scale of 0 to 4 26.

### 2.18 Western blot analysis

To explore the mechanism of macrophage polarization *in vitro* and SCS therapy for stroke *in vivo*. Macrophages were seeded on 6-well plates, 2% FBS culture overnight, and cultured with different neutrophils conditioned media for 15 min. The cells were lysed by RIPA buffer at 4 ℃ for 1 h. Extracted brain tissues were homogenized in 200 μL RIPA buffer, incubated at 4 ℃ for 1 h. All the proteins were collected in the supernatant, measured for concentration, adjusted to the same concentration with loading buffer, and boiled for 10 min. The proteins were separated by gel electrophoresis and transferred to PVDF membrane for visualization by incubation of appropriate antibodies and HRP-conjugated secondary antibody. All antibodies were listed in Table S2. Visualization analysis were performed with chemiluminescence imaging system. Protein expressions were quantified by ImageJ.

### 2.19 RNA-seq and data analysis

After injection of SCS or PBS for 7 days, the infarcted brain tissue was immediately frozen with liquid nitrogen and lysed into RNA-later solution. The RNA sequencing libraries were constructed and sequenced on the BGISEQ-2000 platforms.

### 2.20 SCS tracer test

SCS tracer experiments were performed both *in vivo* and *in vitro*. First, FITC was labeled on SCS (FITC1-1KT, Sigma, United States) according to the manufacturers' instructions. *In vitro*, to explore the interaction between SCS and neutrophils, purified neutrophils stimulated with 1000 ng/mL LPS for 12 h, and then co-cultured with 4 μg/mL FITC-SCS. Flow cytometry and immunofluorescence were performed. *In vivo*, the distribution of SCS in the brain was observed at different time after 150 μL 10 mg/mL FITC-SCS was injected into the orbit. Living imaging (ABL-X8, Tanon, China) and flow cytometry were performed.

### 2.21 Statistical analysis

All results were analyzed using GraphPad. Statistical analysis was done using two-tailed unpaired *t* test for comparisons between two groups and using one-way analysis of variance (ANOVA) or two-way ANOVA for comparison of multiple experimental groups. All statistical data are shown as mean ± SD.

## 3. Results

### 3.1 SCS ameliorates the inflammatory microenvironment in the ischemic hemisphere and promotes angiogenesis in the tMCAO model

SCS was synthesized and its physical properties were thoroughly investigated using Fourier transform infrared spectroscopy (FTIR), gel permeation chromatography (GPC), etc. Analysis of the FTIR spectra revealed the presence of a new absorption peak at 1242 and 816 cm^-1^, which can be attributed to the sulfonate group (Figure S1A). GPC analysis indicated a weight average molecular weight (Mw) of 47600 and a polydispersity index of 1.13 (Figure S1B). Additionally, the sulfur content of SCS was found to be 12.99 ± 0.26% (Figure S1B). Collectively, these findings provide strong evidence that the synthesis of SCS was successful. Moreover, SCS are biosafe at higher concentrations (Figure S2). In particular, SCS could promote the proliferation of RAW264.7 (Figure S2A).

**Figure 1:**
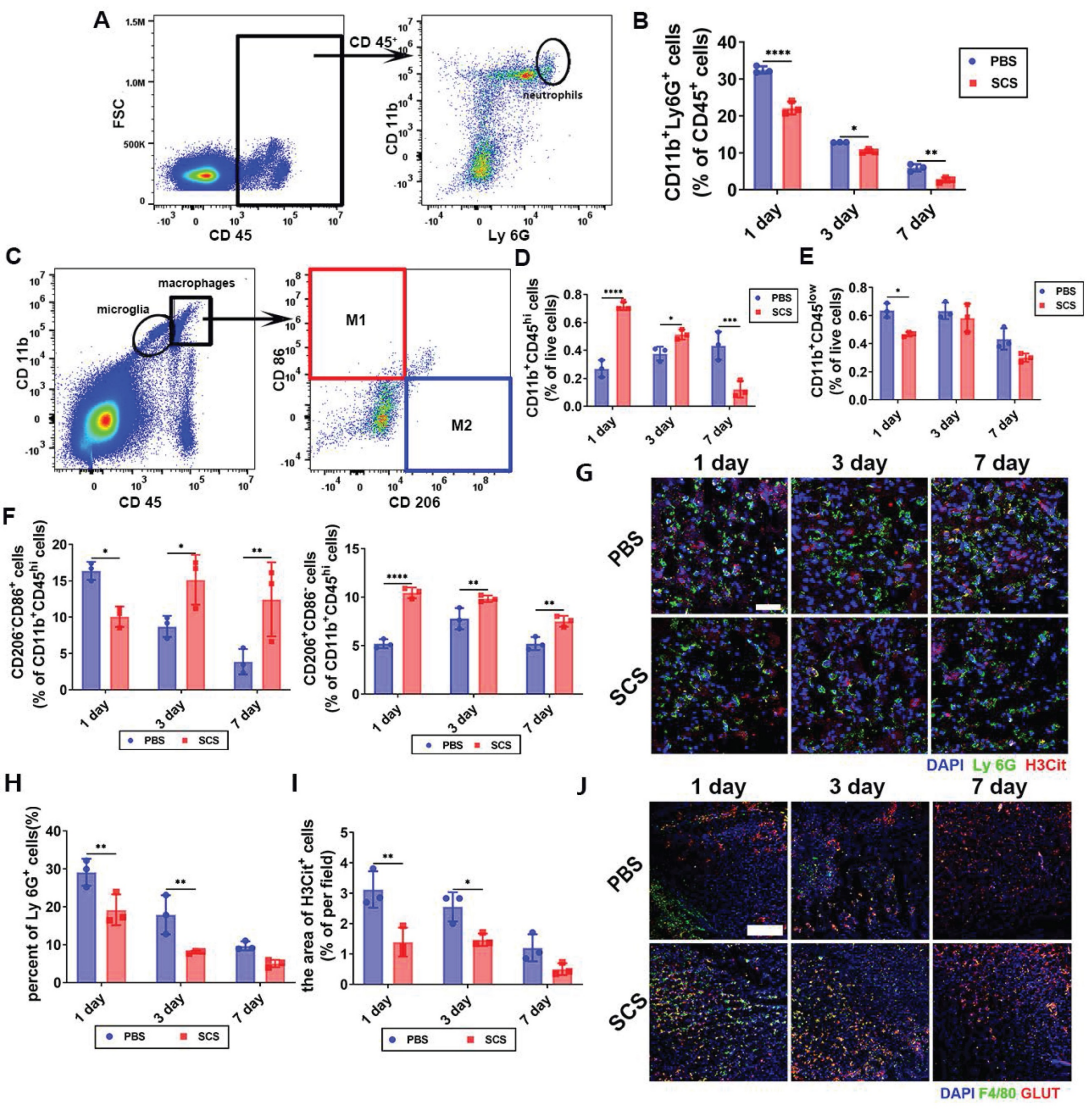
SCS ameliorates ischemic stroke by inhibiting inflammation and promoted angiogenesis. (A and C) Typical flow cytometry and quantification of (B) CD11b^+^Ly 6G^+^ neutrophils, (D) CD11b^+^CD45^hi^ macrophages, (E) CD11b^+^CD45^low^ and (F) CD206^-^CD86^+^ M_1_ macrophages and CD206^+^CD86^-^ M_2_ macrophages after treatment of SCS. Data are shown as mean ± SD (n = 3). (G) Typical immunostained images of Ly 6G (green) and H3Cit (red) and with quantification percentage of (H) Ly 6G^+^ cells and (I) NET. Data are shown as mean ± SD (n = 3). Scar bar was 50 μm. J. Typical immunostained images of F4/80 (green) and GLUT (red). Scar bar was 300 μm. *P < 0.05, **P < 0.01, ***P < 0.001, ****P < 0.0001.

SCS treatment has been found to be effective in regulating immunity by promoting the polarization of macrophages 27. However, this regulatory effect alone is not sufficient to counteract the inflammatory damage caused by acute inflammation. In the case of acute stroke, persistent hyperinflammation can lead to severe damage to brain tissue. Surprisingly, our findings demonstrate that SCS significantly inhibits the infiltration of neutrophils (Figure 1A, B and Figure S3, S4A). On the other hand, macrophages (CD11b^+^CD45^hi^) show a sharp increase during the acute stage (Figure 1C, d and Figure S4B) and microglia (CD11b^+^CD45^low^) only decrease on the first day (Figure 1C, E and Figure S4B). Additionally, SCS treatment promotes the polarization of macrophages towards the M_2_ phenotype (Figure 1C, F and Figure S4C). These findings suggest that SCS not only regulates inflammation by promoting the polarization of macrophages, but also weakens the peripheral inflammatory cascade by inhibiting the infiltration of neutrophils. Immunostaining experiments further demonstrate reduced neutrophil infiltration and increased presence of macrophages during the acute stage (Figure 1G, H and J). Furthermore, SCS treatment is able to clear NETs, thereby inhibiting inflammation (Figure 1G, I) and promoting angiogenesis (Figure 1J and Figure S5). Overall, these results highlight the ability of SCS to reduce the number of neutrophils, while simultaneously recruiting and polarizing macrophages towards the M_2_ phenotype during the acute stage. In essence, it is verified that SCS effectively shortens the acute inflammatory period characterized by neutrophil infiltration, and accelerates the repair period represented by M_2_ macrophages in MCAO mice.

### 3.2 SCS directs the immunomodulation of neutrophils *in vitro*

**Figure 2:**
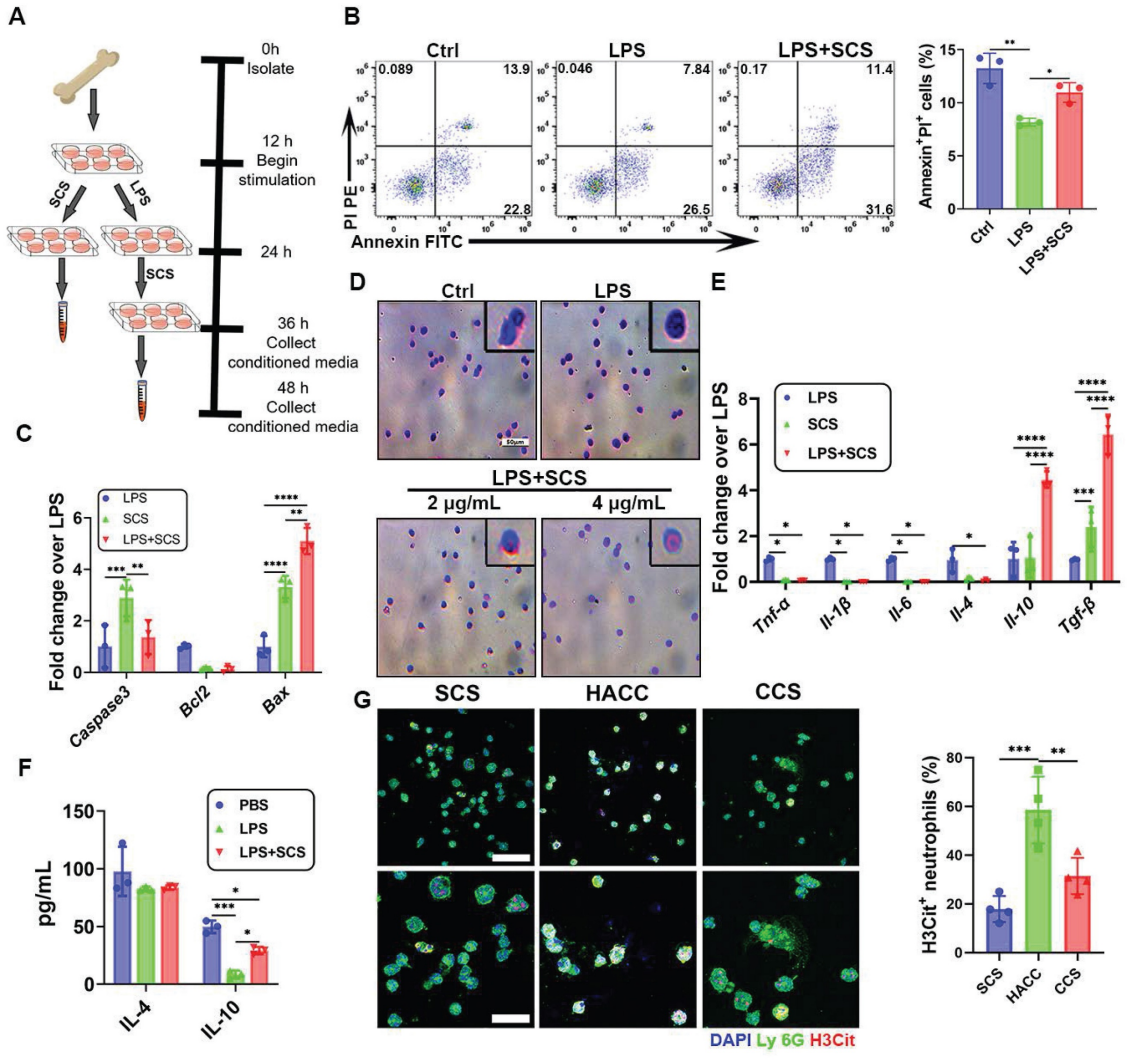
Immunomodulation of neutrophils by SCS *in vitro*. (A) Scheme of obtaining conditioned medium. (B) Typical flow cytometry plots and quantification of neutrophils apoptosis. Data are shown as mean ± SD (n = 3). (C) The genes expressions of neutrophils apoptosis were determined by PCR. Data are shown as mean ± SD (n = 3). (D) Representative images of Wright Giemsa (WG) staining to define nuclear morphology. Scar bar was 50 μm. (E) Gene expressions associated with neutrophil inflammation were performed by qRT-PCR. Data are shown as mean ± SD (n = 3). (F) ELISA of the concentration of IL-4 and IL-10 in neutrophils conditioned media. Data are shown as mean ± SD (n = 3). (G) Typical immunostained images of Ly 6G (green) and H3Cit (red), the second row is a partial enlargement of the first row, and with quantification percentage of NET. Data are shown as mean ± SD (n = 4). Scar bar was 50 μm.*P < 0.05, **P < 0.01, ***P < 0.001, ****P < 0.0001.

To investigate the regulatory effect of SCS on the inflammatory storm, neutrophils were extracted from femurs (Figure 2A). Subsequent flow cytometric analysis revealed that 91.3% of the obtained cells were neutrophils, thus confirming the high purity of the isolated neutrophils (Figure S6A). Cell activity was assessed using SA-*β*-gal and cell cycle assays. Notably, only 17.2% of cells exhibited positivity even after a 48-hour incubation (Figure S6B). Additionally, cell cycle analysis indicated that the majority of cells were in the G0/G1 phase (Figure S6C). These findings collectively illustrate the sustained activity of the isolated neutrophils for up to 48 h.

In the context of innate immune cells, the population of neutrophils plays a pivotal role in shaping the dynamics of pathological environments. An excessive presence of neutrophils can intensify inflammatory responses, highlighting the importance of effectively regulating their abundance for inflammation reduction. Flow cytometry analysis revealed that SCS effectively hindered the delay in neutrophil apoptosis induced by LPS (Figure 2B). This finding was further corroborated by PCR data, which consistently supported the aforementioned conclusion. Gene-level investigations pointed out that upregulation of *Bax* and downregulation of *Bcl2* promote apoptosis of neutrophils in LPS-induced inflammatory environments (Figure 2C). In summation, it can be deduced that SCS holds the capacity to promote neutrophil apoptosis, thereby curbing their infiltration and counteracting the inflammatory surge during the acute inflammatory phase.

The heterogeneity of neutrophils has been observed to have varying regulatory effects on immunity and angiogenesis 12. Wright-Giemsa staining showed that the morphology of nucleus was altered by the presence of 4 μg/mL SCS (Figure 2D), resembling that of N_2_ neutrophils 28. Further investigation into the immune regulation of neutrophils by SCS revealed that it could effectively interact with neutrophils, influencing their behavior as demonstrated through immunofluorescence and flow cytometry analysis (Figure S7A, B). Moreover, gene expression analysis demonstrated that SCS had significant inhibitory effects on the expression of pro-inflammatory genes *Tnf-α*, *Il-1β*, and* Il-6*, in an inflammatory environment, while simultaneously promoting the expression of anti-inflammatory genes *Il-10* and *Tgf-β*, but no effect on *Il-4* (Figure 2E). Consistent with the PCR results, ELISA data indicated that neutrophils in the LPS+SCS group secreted increased levels of IL-10, but exhibited no difference in the secretion of IL-4 (Figure 2F).

Polysaccharides have been found to have a significant scavenging effect on NETs, which are released by stimulated neutrophils during inflammation. The accumulation of NETs can result in vascular obstruction, tissue damage, and prolonged inflammation, which contribute to the progression and worsening of various pathological conditions. In our study, we examined the ability of polysaccharides to clear NETs. Immunofluorescence data showed that except for HACC, polysaccharides were able to effectively clear NETs (Figure 2G). This may be attributed to the electrostatic interaction (Figure S1B). These findings suggest that SCS could play a role in mediating the immune response of neutrophils, inhibiting inflammation, promoting high expression of IL10, and clearing NETs.

### 3.3 SCS regulates angiogenesis of neutrophil *in vitro*

**Figure 3:**
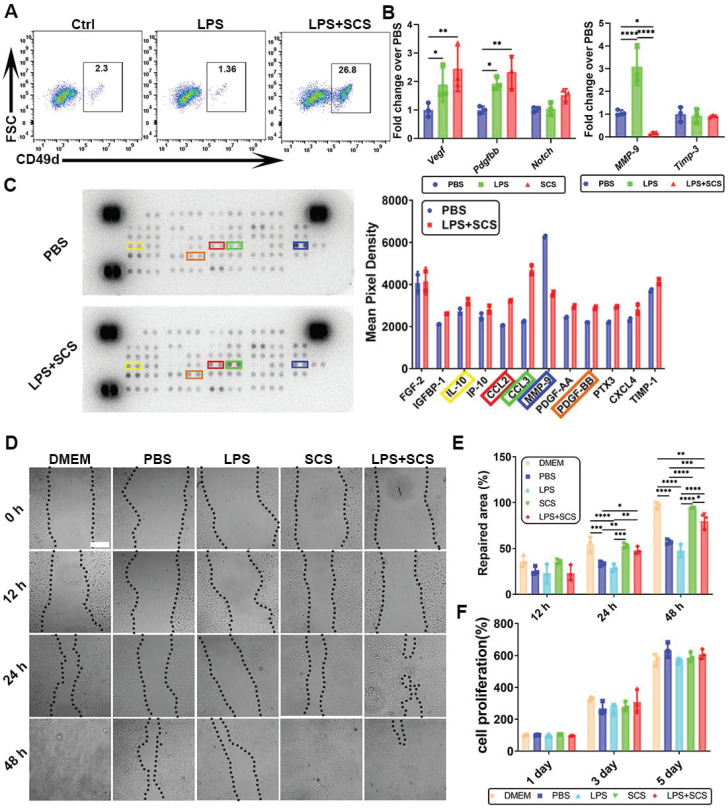
Effect of SCS on angiogenesis of neutrophils *in vitro*. (A) Typical flow cytometry of CD49d^+^ vessel-related neutrophil subsets. (B) Typical expression of genes associated with angiogenesis in neutrophils. Data are shown as mean ± SD (n = 3). (C) Mouse cytokine array of different neutrophils stimulated by SCS and with quantification typical vascular factor. (D) Typical image of the migration of HUVECs, and (E) with quantification of repaired area. Data are shown as mean ± SD (n = 3). Scale bar is 200 μm. (F) Quantification of HUVEC proliferation after incubation with different neutrophil conditioned medium. Data are shown as mean ± SD (n = 3). *P < 0.05, **P < 0.01, ***P < 0.001, ****P < 0.0001.

The study found that neutrophils stimulated by SCS exhibited a distinct phenotype, with a CD49d^+^ cell subset (Figure S8), known to be conducive to angiogenesis 29. Flow cytometry analysis demonstrated that SCS can induce the production of CD49d^+^ cell subsets in inflammatory environments, indicating the ability of SCS to drive neutrophils towards angiogenic phenotypes (Figure 3A). Gene expression analysis further confirmed that SCS significantly up-regulated the expression of *Vegf* and *Pdgfbb* in neutrophils (Figure 3B). Additionally, SCS was observed to inhibit the expression of MMP-9, an enzyme involved in extracellular matrix degradation and blood vessel leakage (Figure 3B). Consistent with gene expression data, Proteome Profiler analysis revealed that SCS promoted the secretion of IL-10 and PDGFBB, while inhibiting the secretion of MMP-9 (Figure 3C). The response of endothelial cells to the microenvironment directly affects angiogenesis. Therefore, the impact of neutrophils stimulated by SCS on endothelial cell proliferation and migration was assessed. Interestingly, SCS-stimulated neutrophils significantly enhanced endothelial cell migration (Figure 3D, E), but had no effect on proliferation (Figure 3F). In summary, these findings demonstrate that SCS stimulation of neutrophils can lead to the release of angiogenic-related factors and promote endothelial cell migration.

### 3.4 Neutrophils stimulated by SCS mediate macrophage polarization via IL-10-induced Stat3 signaling pathway

**Figure 4:**
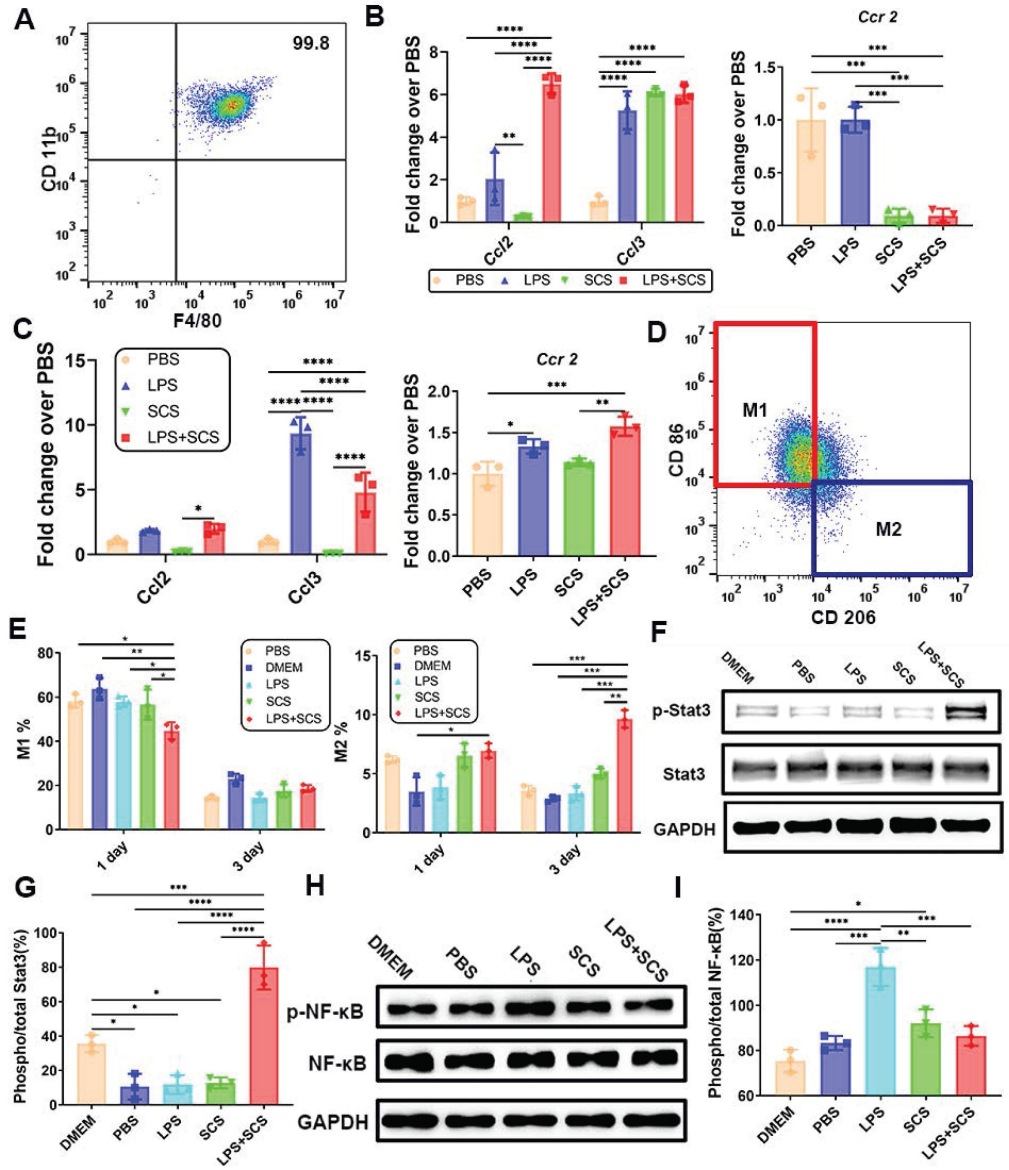
The infiltration of macrophages is promoted by CCL2/3-CCR2, and the polarization of macrophages is induced by IL-10-Stat3. (A) Purity of isolated mouse peritoneal macrophages identified by flow cytometry. (B) Genes expressions of neutrophils of neutrophils. Data are shown as mean ± SD (n = 3). (C) Genes expressions of macrophages. Data are shown as mean ± SD (n = 3). (D) Typical flow cytometry and (E) quantification of CD206^-^CD86^+^ M_1_ macrophages and CD206^+^CD86^-^ M_2_ macrophages. Data are shown as mean ± SD (n = 3). (F) Western blots (G) with quantification and the relative level of the phosphorylated Stat3. Data are shown as mean ± SD (n = 3). (H) Western blots analysis (I) with quantification and the relative level of the phosphorylated NF-κB after treating with the conditioned medium. Data are shown as mean ± SD (n = 3). *P < 0.05, **P < 0.01, ***P < 0.001, ****P < 0.0001.

To investigate the reason why macrophages can be recruited rapidly in the acute stage, we obtained highly pure mouse peritoneal macrophages (Figure 4A). Infiltration of leukocytes and lymphocytes is facilitated by chemokines CCL2 and CCL3. Proteome Profiler data have demonstrated that SCS promotes the release of CCL2 and CCL3 from neutrophils (Figure 3C). Consistent results were obtained at the genetic level through PCR analysis, where Ccr2, the receptor for Ccl2, showed down-regulation in neutrophils (Figure 4B) and up-regulation in macrophages (Figure 4C). This suggests that CCL2/3 released by neutrophils stimulated by SCS in the acute stage and binds to the CCR2 receptor on macrophages, thereby promoting macrophage infiltration.

The heterogeneity of macrophages has been extensively studied, and M_2_ macrophages are generally recognized to play a beneficial role in tissue repair and regeneration. The infiltration of macrophages directly influences tissue repair. Additionally, there is evidence suggesting that SCS contributes to the polarization of macrophages 23. However, the precise mechanism by which SCS mediates macrophage polarization remains unclear. In our study, we cultured peritoneal macrophages using different neutrophil conditioned media. Through flow cytometry analysis, we observed that neutrophils stimulated by SCS inhibited the polarization of macrophages towards M_1_ in an inflammatory environment on day 1 (Figure 4D, E, and Figure S9). Furthermore, SCS significantly regulated macrophage polarization towards M_2_ (Figure 4F, Figure S9). This effect may be attributed to the presence of programmed apoptotic neutrophil fragments, which favor the M_2_ polarization of macrophages 30.

Overall, our findings suggest that SCS-induced polarization of macrophages could potentially contribute to tissue repair and regeneration. Further investigations are required to fully understand the molecular mechanisms underlying this process. According to the data from PCR, ELISA, and Proteome Profiler, it was observed that neutrophils, when stimulated by SCS in an inflammatory environment, release a large amount of IL-10 instead of IL-4 (Figure 2E, F and Figure 3B). Moreover, western blot analysis revealed that the phosphorylation of Stat3 in macrophages was activated in the LPS + SCS group (Figure 4F, G). Consequently, it can be inferred that neutrophils stimulated by LPS with SCS mediate macrophage polarization through the IL-10-induced Stat3 signaling pathway. Additionally, the western blot analysis demonstrated that the neutrophils conditioned medium, when stimulated by SCS, significantly inhibits the phosphorylation of NF-κB in macrophages (Figure 4H, I). This suggests that the neutrophils conditioned medium, under SCS stimulation, possesses the ability to effectively suppress inflammation in macrophages.

### 3.5 The efficacy of SCS in the treatment of ischemic stroke

**Figure 5:**
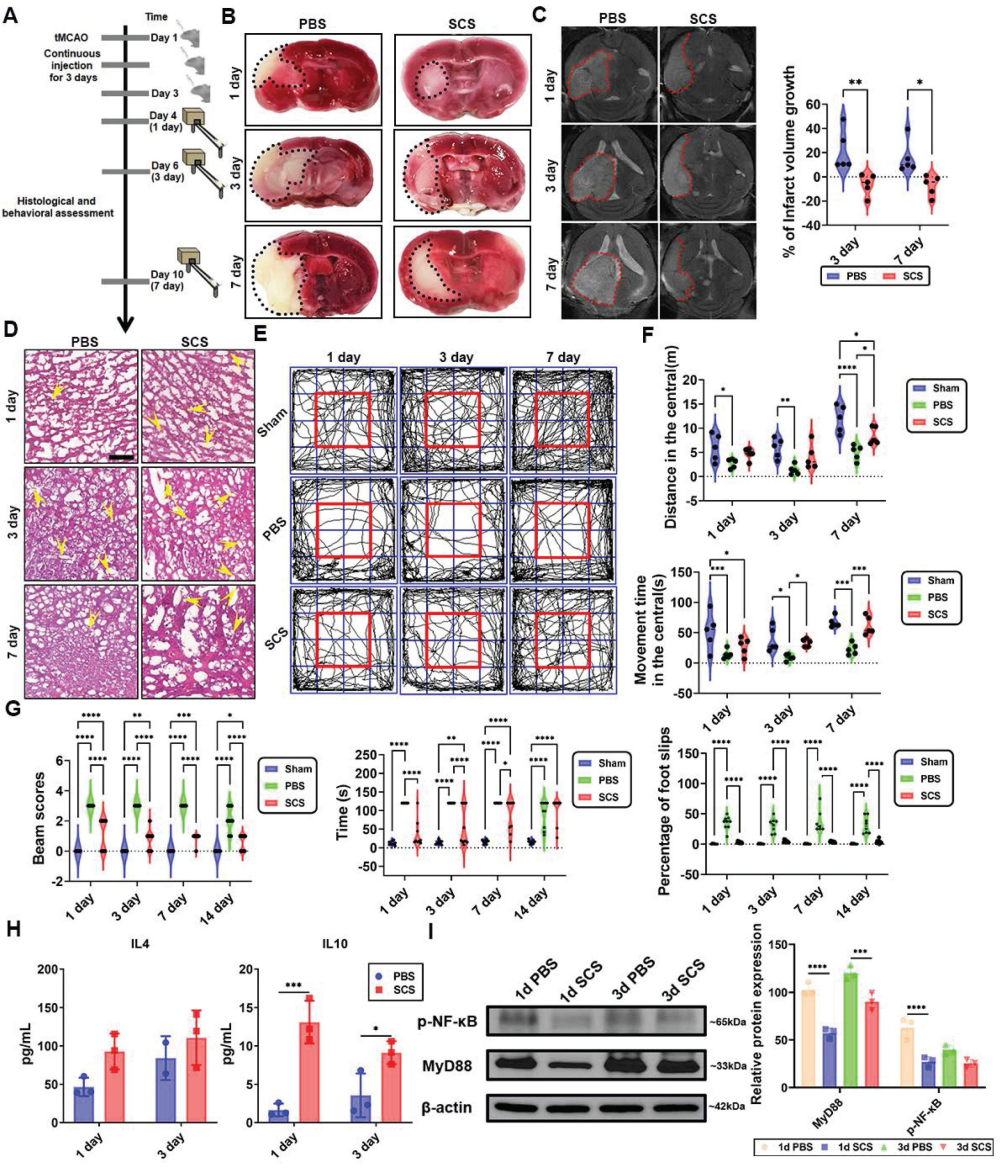
The efficacy of SCS in the treatment of ischemic stroke. (A) The schematic illustration of stroke surgery and experimental design. (B) Typical coronal brain sections were stained with TTC. (C) T2-weighted magnetic resonance (MR) images at day 1, 3, and 7 after treatment of SCS with quantification of infarction volume. Data are shown as mean ± SD (n = 5). (D) Typical images of H&E staining. The yellow arrows point to vessels. Scale bar is 50 μm. (E-F) Representative track plots during a 15 min open field test (E), movement distance during the open field test (F), and movement time spent during the 15 min open field (F). Data are shown as mean ± SD (n = 5). (G) Beam balance score of I/R mice treated with SCS at day 1, 3, 7, and 14, the time to cross the beam and the number of foot slips were analyzed. Data are shown as mean ± SD (n = 9). (H) ELISA analysis of IL-4 and IL-10 in brain tissue. Data are shown as mean ± SD (n = 3). (I) Western blots analysis and the relative level of the MyD88, and p-NF-κB after treating with SCS. Data are shown as mean ± SD (n = 3). *P < 0.05, **P < 0.01, ***P < 0.001, ****P < 0.0001.

The efficacy of SCS in treating ischemic stroke was further assessed by utilizing the tMCAO model (Figure 5A). Extensive histology and motor abilities evaluations were conducted to validate the brain-protective effects of SCS *in vivo*. The therapeutic impact of SCS was found to be dependent on dosage and times, with the lowest mortality rate observed at 1500 μg (150 μL 10mg/ mL) SCS for three consecutive days, and higher concentrations of SCS at 3000 μg (150 μL 20 mg/mL) resulted in increased mortality rates (Figure S10A). Furthermore, injections of 1500 μg SCS for one day did not improve stroke mortality (Figure S10B).

After reperfusion, a MCAO mouse was administered 10 mg/mL FITC-conjugated SCS via orbital injection. Real-time fluorescence imaging revealed the accumulation of SCS at 1 h, with maximum accumulation observed at 6 h after reperfusion. However, at 24 h, fluorescence was barely observed (Figure S11A). Flow cytometry data from 1, 6, 12, and 24 h after orbital injection of SCS showed that the peak value of FITC was observed at 6 h in both blood (Figure S11B) and brain tissue (Figure S11C). To study the residence time of SCS in different brain regions, FITC content in the brain was observed at 12 h, indicating significantly higher content in the infarct side compared to the contralateral brain (Figure S11D). Furthermore, flow cytometry analysis revealed that the main cell types carrying FITC fluorescence were neutrophils, with some being macrophages (Figure S11E, F). These findings demonstrate that SCS can be transported by leukocytes to the infarct area during inflammatory storms and remain active for 24 h.

SCS treatment, as observed in brain tissues stained with 2,3,5-triphenyl tetrazolium chloride (TTC), resulted in a significant reduction in the infarct area when compared to the PBS group (Figure 5B, Figure S12A). Additionally, MRI analysis confirmed that SCS treatment effectively attenuated infarct volume expansion (Figure 5C). Pathological examination revealed that the PBS group exhibited cytoplasm vacuolation, cytoplasm shrinkage, and fewer blood vessels in the cerebral cortex (Figure 5D, Figure S12B). To assess the improvement in locomotor function, the open field and balance beam test were conducted on days 1, 3, and 7 after the administration of SCS. In the PBS group, motor ability and cognitive ability were severely impaired following ischemia reperfusion (I/R) (Figure 5E-G, Figure S10C, D). Conversely, the SCS group showed increased distance and movement time in the central area of the open field test (Figure 5E, F), indicating improved locomotor and cognitive competence. Moreover, balance beam scores were significantly higher in the SCS group (Figure 5G). The administration of SCS also led to a notable reduction in passing time and slip steps during the balance beam test (Figure 5G), suggesting significant improvements in balance function in the SCS group.

The canonical IL-4-mediated Stat6 or IL-10-mediated Stat3 signaling pathways have been identified to play a crucial role in macrophage polarization 31. In the peripheral blood, IL-4 secretion was observed to be high on day 1 and day 3, while IL-10 was predominantly secreted on day 1 (Figure S13). However, in the brain, no significant difference in IL-4 levels was observed, but there was a notable increase in IL-10 (Figure 5H). Based on the Western blotting results (Figure 4F), it can be concluded that macrophage polarization in the brain following stroke primarily occurs through IL-10-mediated Stat3 signaling pathways. NF-κB, a classical immunomodulatory signaling pathway, has been shown to mitigate brain infarct volume 4,32. In the ischemic hemisphere, SCS treatment significantly downregulated P-NF-κB and upstream MyD88 signaling pathways (Figure 5I). These findings suggest that inhibiting inflammation via the MyD88-NF-κB signaling pathway helps ameliorate the local microenvironment.

### 3.6 RNA-seq analysis of stroke mice treated with SCS

**Figure 6:**
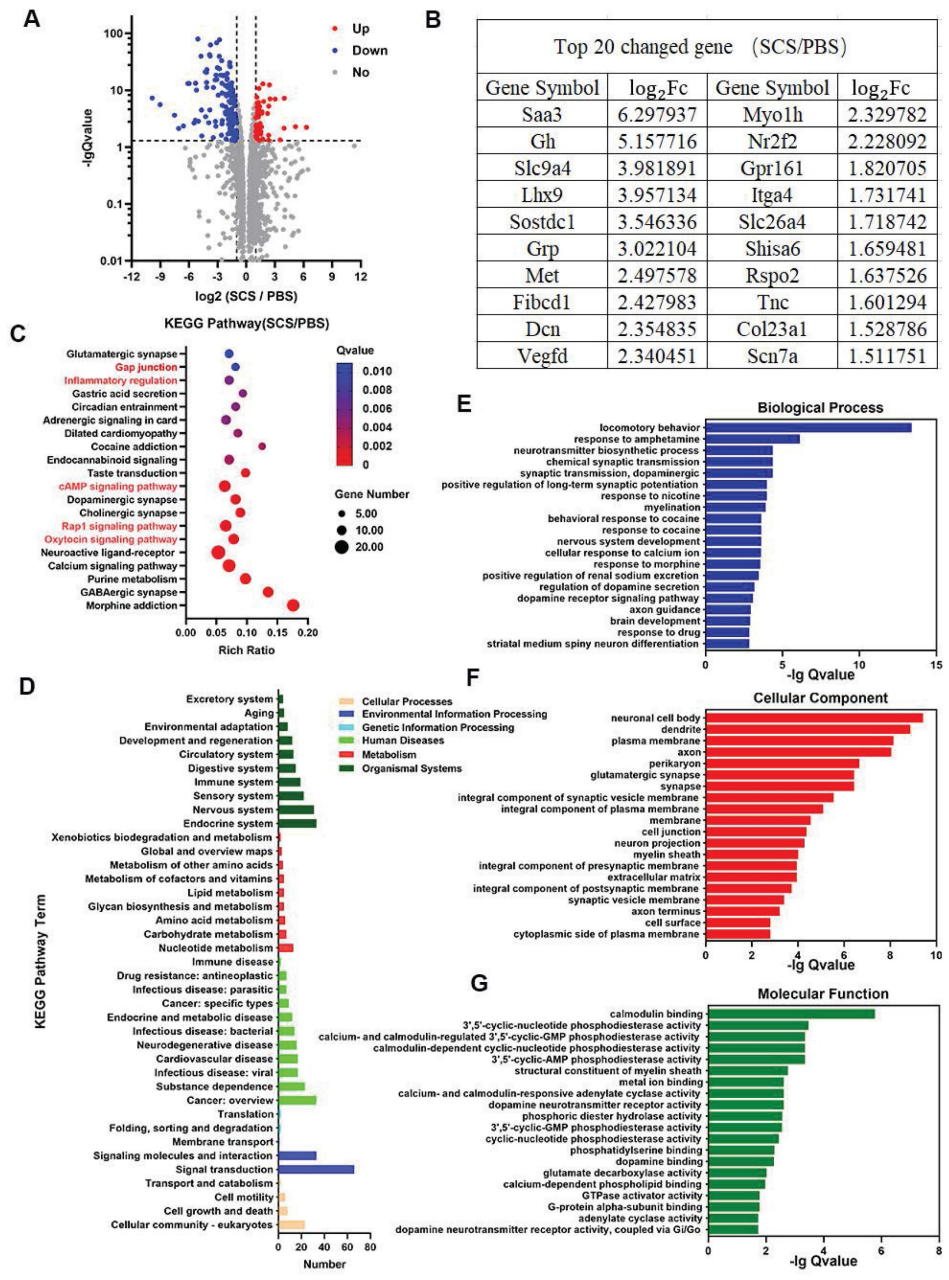
RNA-seq analysis of stroke mice treated with SCS. (A-B) Significant gene expression changes in genes (A), and a list of top 20 upregulated genes and corresponding log2Fc (B). (C) KEGG pathway enrichment analysis of SCS/PBS comparison. (D) KEGG pathway classification analysis of SCS/PBS comparison. (E-G) Significant GO terms of associated biological processes (E), cellular components (F), and molecular functions from differentially regulated genes (G).

According to our assessment of gene expression using RNA-seq in brain tissue at day 7 after SCS administration, we observed significant changes (Figure 6). Under the condition of | log2 F_c_ | > 1 and Q value < 0.05, there was a total of 194 differentially expressed genes (DEGs) out of 17,866 gene transcripts. Among the DEGs, 54 genes were up-regulated while 140 genes were down-regulated (Figure 6A). Based on these findings, we focused on the top 20 genes with the highest changes among the DEGs. Interestingly, several of these genes were found to be associated with critical aspects of brain growth and development (*Gh, Lhx9, Dcn*), immune regulation (*Sostdc1*), and angiogenesis (*Vegfd*) (Figure 6B). These findings indicate that stroke recovery following SCS treatment might be linked to processes involving immune regulation, angiogenesis, and ECM reconstruction.

According to the Kyoto Encyclopedia of Genes and Genomes (KEGG) pathway enrichment analysis, several pathways exhibit a significant enrichment. These include the oxytocin signaling pathway, Rap1 signaling pathway, cAMP signaling pathway, inflammatory regulation, and gap junction (Figure 6C). The oxytocin signaling pathway and inflammatory regulation are involved in oxidative stress and immune regulation of immune cells. The Rap1 signaling pathway is also implicated in inflammatory regulation. Additionally, the cAMP signaling pathway and gap junction are involved in vascular remodeling and the protection of vascular endothelial cells, leading to a reduction in vascular permeability and the preservation of the BBB. KEGG pathway classification analysis further reveals a higher number of differential genes in the immune system, cardiovascular disease, and cell growth and death (Figure 6D).

In order to investigate the relationship of differentially expressed genes (DEGs), a gene ontology (GO) enrichment analysis was conducted on biological process, cellular component, and molecular function (Figure 6E-G). The top 20 significant GO terms (based on Q value) were identified. Notably, GO terms related to motor behavior, changes in the cell membrane, and protein interactions were significantly enriched. These terms included locomotory behavior, cellular response to calcium ion, and brain development within the biological process category, as well as plasma membrane, integral component of plasma membrane, membrane, cell junction, extracellular matrix, and cell surface within the cellular component category. Additionally, notable GO terms within the molecular function category included phosphatidylserine binding and G-protein alpha-subunit binding. Overall, these findings suggest that SCS therapy has the potential to improve stroke outcomes by modulating motor behavior, altering membrane protein expression, and ECM remodeling in mice.

### 3.7 SCS treats ischemic diseases by attenuating the inflammatory cascade

Ischemic stroke is a major cerebrovascular disease associated with inflammation. SCS can play a therapeutic role by regulating inflammation. However, it needs to be confirmed that SCS influence the inflammatory cascade by regulating neutrophils. Therefore, we implanted GelMA hydrogels loaded with neutrophil conditioned media in a mouse ischemic hind limb model, which can prove the universality of SCS mediating neutrophils to regulate inflammatory microenvironment and promote angiogenesis.

The physical properties of GelMA were investigated using Fourier transform infrared spectroscopy (FTIR), nuclear magnetic resonance (NMR), and scanning electron microscopy (SEM). Upon analysis, a new absorption peak at 836 cm^-1^ and the ^1^H NMR spectrum at 5.4 and 5.6 ppm were observed, indicating the successful grafting of double bonds onto the gelatin (Figure S14A, B). Furthermore, SEM images revealed significant differences in surface morphologies and porous structures of GelMA hydrogel after crosslinking (Figure S14C). These findings contribute to a better understanding of GelMA hydrogel and its potential applications.

**Figure 7:**
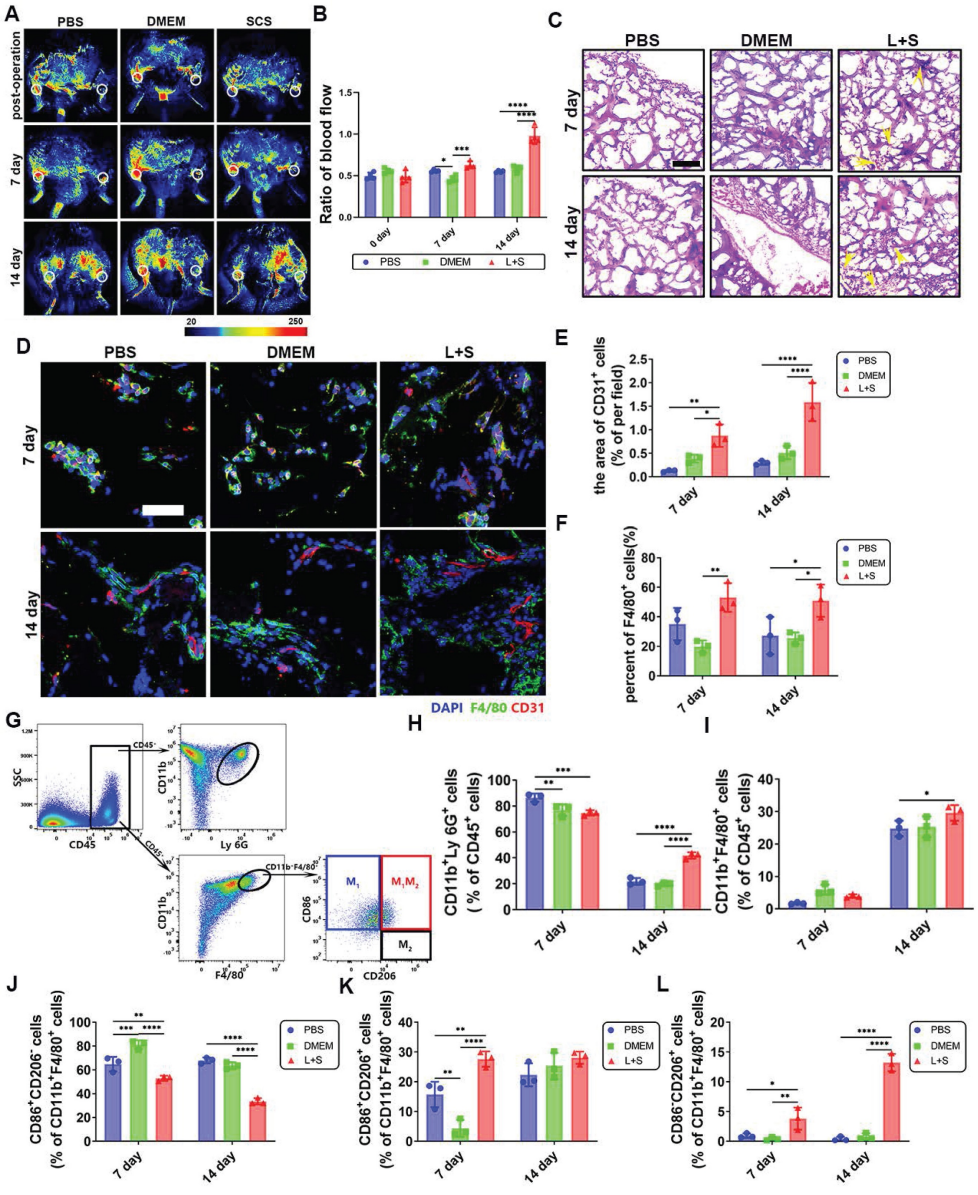
Vascular and inflammatory regulation of SCS in mouse ischemic hind limb. (A) Typical image of a blood flow. The white circle represents the surgical site or corresponding opposite position. (B) Quantitative analysis of ratio blood flow. Data are shown as mean ± SD (n = 4). (C) Typical image of H&E staining. The yellow arrows point to the vessel. Scale bar is 200 μm. (D-F) Typical immunostained images (D) of CD31 (red) and F4/80 (green) and quantification (E) the area of CD31 endothelium and (F) F4/80^+^ macrophages after implantation in ischemic limb. Data are shown as mean ± SD (n = 3). Scale bar is 50 μm. (G) FACS plots of immune cell populations isolated for implantation in ischemic limb. (h-i) Quantification of (H) CD11b^+^Ly 6G^+^ neutrophils and I) CD11b^+^F4/80^+^ macrophages after implantation in ischemic limb. Data are shown as mean ± SD (n = 3). (J-L) Quantification (J) of CD206^-^CD86^+^ M_1_ macrophages, (K) CD206^+^CD86^+^ macrophages and (l) CD206^+^CD86^-^ M_2_ macrophages. Data are shown as mean ± SD (n = 3). *P < 0.05, **P < 0.01, ***P < 0.001, ****P < 0.0001.

Our study aimed to investigate the effect of SCS in the treatment of ischemic diseases related to inflammation. We implanted GelMA hydrogels in a mouse ischemic hind limb model. Laser Speckle Contrast Imaging showed that the blood perfusion in the surgical location was reduced by approximately 50% after surgery (Figure 7A, B). The blood perfusion gradually increased over time. Notably, on day 7, the hydrogels containing neutrophil conditioned medium induced by SCS in an inflammatory environment (L+S) showed higher levels of perfusion. Furthermore, the collateral circulation perfusion significantly recovered in the L+S group on day 14 (Figure 7A, B).

To further investigate how SCS stimulated neutrophils accelerate angiogenesis, we conducted histological and pathological evaluations. H&E staining revealed the presence of microvascular structures in the L+S group on day 7, and an increased number of erythrocytes were observed on day 14 (Figure 7C). Immunostainings of CD31^+^ cells showed a larger area of expression in the L+S group with LPS stimulation (Figure 7D, E), which is consistent with the findings from the H&E staining. Additionally, F4/80^+^ macrophages were found to cluster noticeably in the L+S group (Figure 7D, F). Overall, our results suggest that SCS, in an inflammatory milieu, has a positive effect on blood perfusion, angiogenesis, and the recruitment of macrophages. These findings provide valuable insights into the potential therapeutic applications of SCS in ischemic diseases related to inflammation.

The inflammatory response was analyzed using flow cytometry (Figure 7G and Figure S15-S17). Surprisingly, on day 7, the L+S group exhibited a lower infiltration of neutrophils (Figure 7H, Figure S17A), whereas the number of macrophages significantly increased on day 14 (Figure 7I, Figure S17B). Due to the heterogeneity of macrophages, it is widely accepted that M_1_ macrophages promote inflammation while M_2_ macrophages are involved in tissue regeneration, with phenotypes capable of transitioning in the local microenvironment. Consequently, we analyzed the macrophage phenotype within the implant. Flow cytometry data revealed that SCS remarkably inhibited M_1_ macrophages (CD86^+^CD206^-^) (Figure 7J, Figure S17C), promoted the double positive phenotype (Figure 7K, Figure S17C), and facilitated the M_2_ macrophages (CD86^-^CD206^+^) (Figure 7L, Figure S17C). Aligning these findings with the increased macrophage count, we concluded that a greater number of recruited macrophages were being converted into the M_2_ phenotype within the L+S group. In summary, these results provide evidence that SCS reduces neutrophil infiltration, recruits and polarizes macrophages towards the M_2_ phenotype through neutrophil regulation, thereby shortening the acute inflammatory period and accelerating the repair period.

## 4. Discussion

Hypoxia is a significant trigger for inflammation, oxidative stress, and apoptosis in cases of ischemic stroke. During acute stroke, the rescue of the penumbra relies on rapid restoration of vascularization and improvement of the inflammatory microenvironment, which involves various cells and factors including immune cells, blood vessels, and nerve-related cells 33,34. Immune regulation has been shown to improve the prognosis of patients 35. Our previous study demonstrated that N_2_ neutrophils have the ability to inhibit inflammation and promote angiogenesis 12. By effectively regulating the behavior of neutrophils, inflammation can be suppressed at its origin. However, the mechanism for attenuating inflammatory initiation remains poorly understood. Our current study focuses on GelMA hydrogel prepared with neutrophil conditional medium, which has shown promising results in inhibiting the early hyperinflammatory state. This is evidenced by a decrease in neutrophil infiltration and improved vascular perfusion in a mouse model of ischemic hind limb (Figure 7). Therefore, SCS offer a potential therapeutic strategy for ischemic diseases, as they hold the potential to weaken inflammatory initiation and promote vascular regeneration.

The heterogeneity of inflammatory cells plays a crucial role in regulating tissue regeneration. Previous studies have highlighted the role of N_2_ neutrophils and M_2_ macrophages in promoting angiogenesis, bone regeneration, and stroke recovery 16,36,37. Neutrophils have been recognized as key players in regulating inflammation microenvironment and shaping the immune response during the acute inflammatory phase of stroke. What's more, the microenvironment for the survival of blood vessels and nerves has often been overlooked. In our study, we aimed to investigate the effects of SCS on modulating the inflammatory response and its impact on tissue regeneration. Our findings demonstrate that SCS promotes apoptosis of neutrophils, resulting in reduced infiltration of inflammatory cells and preventing the exacerbation of inflammation. Caspase3 and Bcl2/Bax are two key pathways in the apoptosis process 38,39. Our results demonstrated that neutrophils can phagocytose SCS and initiate neutrophilic reprogramming, mainly through the Bcl2/Bax apoptosis pathway (Figure 2C), which promotes neutrophilic apoptosis in a non-Caspase3-dependent manner.

As for the regulation of the inflammatory cascade, PCR analysis further confirmed that SCS down-regulates proinflammatory genes in neutrophils and promotes the expression and release of IL-10. Interestingly, we observed that under SCS stimulation, neutrophils promote the polarization into M_2_ macrophages through the IL-10-mediated Stat3 signaling pathway, instead of the conventional IL-4 pathway. Moreover, SCS exhibits strong electronegativity, enabling it to scavenge positive inflammatory factors and chemokines, which are danger signals in the inflammatory response 40. The removal of these early danger signals contributes to the attenuation of inflammatory storms 41,42. *In vivo* experiments showed that SCS can alleviate inflammation by shortening the acute inflammatory period, as evidenced by a decrease in the number of neutrophils, and accelerating the repair period, as indicated by an increase in M_2_ macrophages. These results indicate that neutrophils, as the fastest messengers of the inflammatory response, are influenced by SCS derived from the inflammatory origin. This, in turn, affects the inflammatory microenvironment and attenuates the acute inflammatory phase. Further research is warranted to elucidate the precise mechanisms underlying these effects and explore the therapeutic potential of SCS in inflammatory conditions.

Suppressing the inflammatory response is advantageous in promoting tissue repair and regeneration, particularly in terms of angiogenesis 43,44. While moderate inflammation aids in debris clearance and inhibiting bacterial infection, excessive inflammation can worsen tissue necrosis. Precise regulation of inflammation is crucial for tissue regeneration. Additionally, SCS can be combined with various growth factors and cytokines to sustain their activity, such as VEGF 45. Neutrophils activated by SCS specifically produce the CD49d^+^ cell subset during inflammatory conditions, which releases VEGF and PDGFBB to facilitate endothelial cell migration 46. Furthermore, the formation of thrombus serves as a trigger for recurrent strokes. Inflammation activates neutrophils, prompting them to release NET 47, thereby promoting thrombosis 48,49. Surprisingly, SCS effectively neutralizes NET due to its strong electronegativity. These findings indicate that SCS significantly contributes to preventing the recurrence of cardiovascular and cerebrovascular diseases.

The extracellular matrix of the brain, predominantly consisting of glycosaminoglycans 50. SCS, glycosaminoglycan-like polysaccharide, can be enriched in the ischemic hemisphere through the BBB. In addition, MMP-9, known for its ability to degrade extracellular matrix and promote angiogenesis 51, can also increase vascular permeability and disrupt the BBB when present in excessive amounts 52. In our experiment, we observed that MMP-9 was significantly inhibited at the gene and protein levels, suggesting the promotion of extracellular matrix reconstruction in this region. IL-10, acting through the IL-10-STAT3 signaling pathway, was found to facilitate the polarization of macrophages towards the M_2_ phenotype in the ischemic hemisphere. On the contrary, NF-κB activation, induced by various inflammatory factors, can exacerbate inflammation 53. However, SCS effectively inhibited the MyD88-p-NF-κb signaling pathway in the ischemic hemisphere, thus improving the pathological microenvironment associated with stroke. Furthermore, our RNA sequencing results revealed up-regulation of the *Fibcd1*, *Nr2f2*, and *Gpr161* genes, which are involved in nerve repair processes. Amplification of nerve-related signaling pathways was also evident in our KEGG and GO analyses. Based on this evidence, we propose that SCS may promote nerve recovery following stroke. The close interaction between the nervous system and the immune system, facilitated by various mechanisms, plays a pivotal role in the recovery process after ischemic brain injury 54.

## 5. Conclusions

According to our research, we have developed a groundbreaking approach to mitigate the immune cascade by modulating neutrophil behavior, effectively suppressing inflammation from its source. Our strategy involves the SCS, which has demonstrated the ability to induce neutrophil apoptosis, thereby curbing the occurrence of inflammation and reducing the acute inflammatory period. Furthermore, neutrophils stimulated by SCS have been found to enhance the polarization of macrophages towards the M_2_ phenotype, hastening the repair period. Remarkably, SCS also stimulates neutrophils to generate a vascular-related subset, leading to angiogenesis, a process similar to the action of TGF-β. These findings hold promising implications for suppressing inflammatory storms in cardiovascular and cerebrovascular diseases and provide effective strategies for curbing secondary injuries. Crucially, our approach relies on non-exogenous growth factors and cells, highlighting its remarkable potential for clinical translatability in the treatment of major organ injuries. In conclusion, our novel strategy offers an effective means to combat inflammation and represents a significant advancement in the field of medical treatment.

## Supplementary Material

Supplementary figures and tables.

## Abbreviations

SCS: sulfated chitosan
MCAO: middle cerebral artery occlusion
RT-PCR: Real-Time Quantitative Reverse Transcription
BBB: blood-brain barrier
I/R: ischemic/reperfusion
GAG: glycosaminoglycan
MMP 9: Matrix metalloproteinase 9
GelMA: methacrylated gelatin
LPS: lipopolysaccharide
net: neutrophil extracellular trap
HACC: chitosan quaternary ammonium salt
CCS: carboxylation chitosan
MRI: magnetic resonance imaging
