# Supplementary material for: Sulfated chitosan directs the recovery of ischemic stroke by attenuating the inflammatory cascade
Source: Theranostics. 2025 Apr 28;15(12):5870–89. doi: 10.7150/thno.111681 (PMC12068298; doi:10.7150/thno.111681)
Supplement: Supplementary file 1 — Supplementary figures and tables. [file thnov15p5870s1.pdf]

A

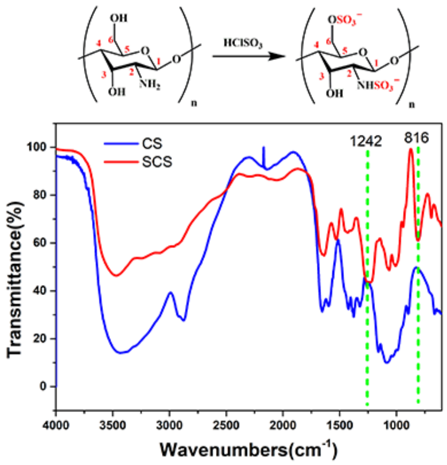

B

Sample	Mn(Da)	Mw(Da)	Sulfur content(%)
SCS	42000	47600	12.99±0.26

Sample	SCS	HACC	CCS
Zata-potential (mV)	-51.9±2.1	59.3±0.9	-45.6±1.7

Figure S1. Characterization of SCS

(A) The structural formula and Fourier Transform Infrared (FTIR) of the CS and SCS. (B) The gel permeation chromatography (GPC), sulfur content of the SCS, and the Zeta-potential of different carbohydrate polymers.

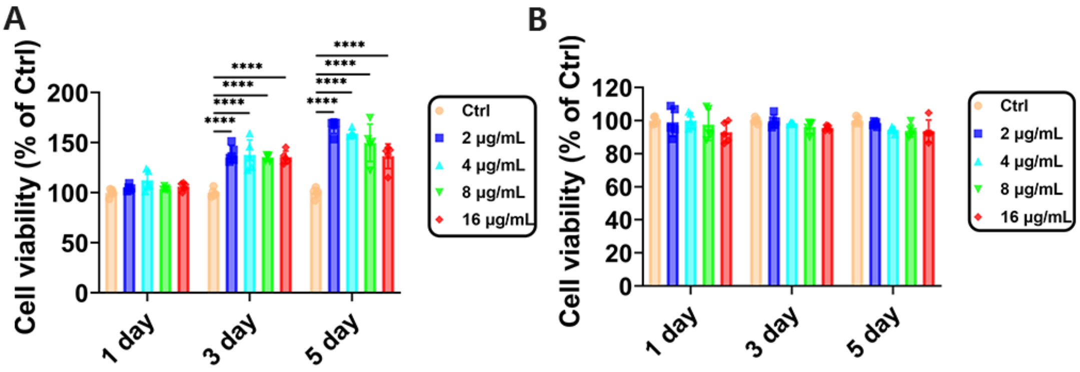

Figure S2. The biosafety of SCS against RAW 264.7 (A) and HUVECs (B).

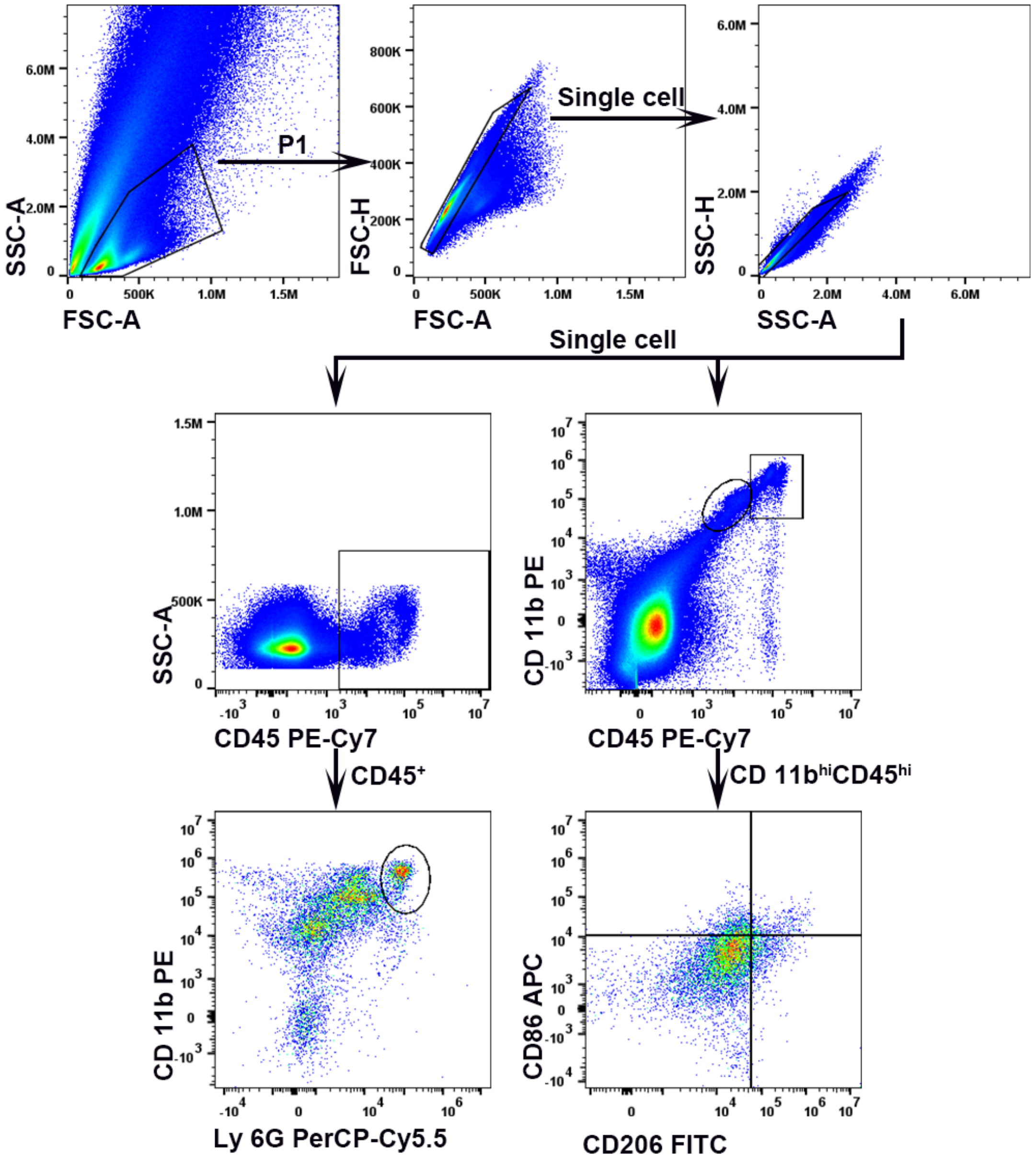

Figure S3. Strategy of sorting neutrophils and macrophages in ischemic stroke.

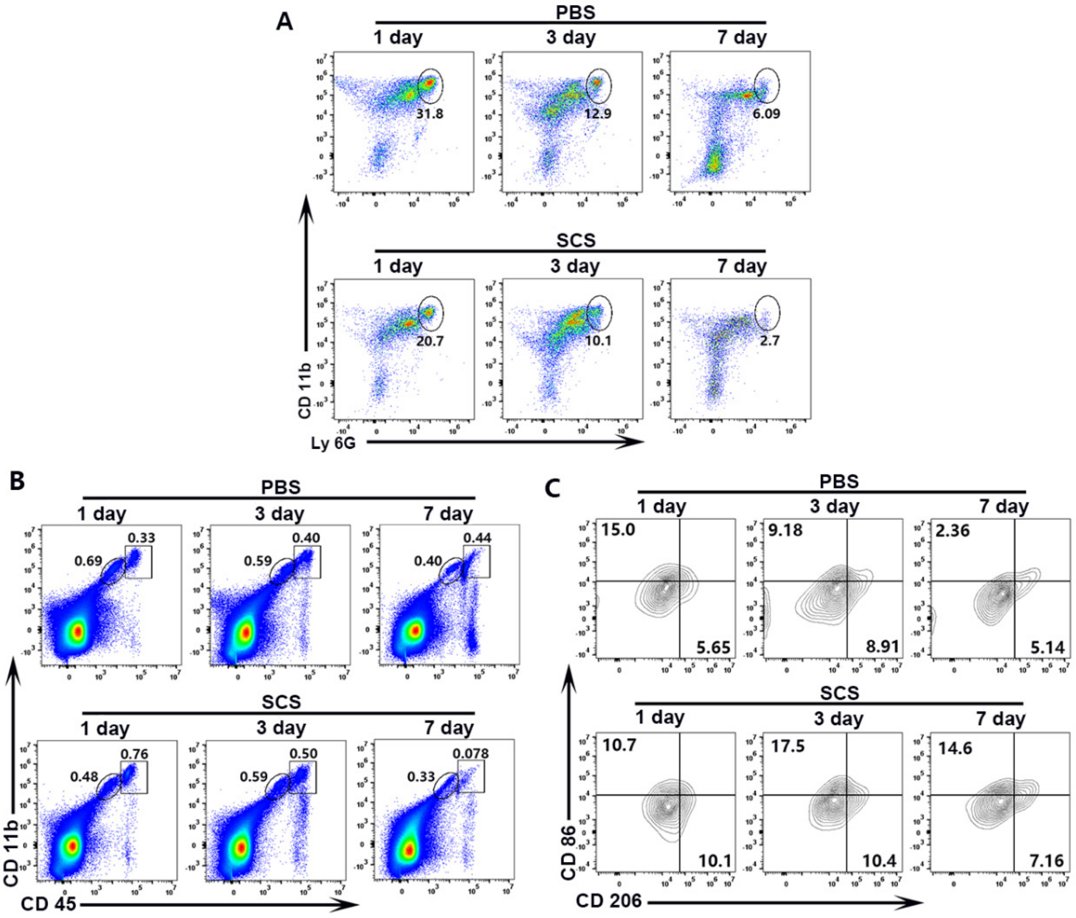

Figure S4. Representative flow cytometry plots in the tMCAO model.

(A-C) Representative flow cytometry plots of (A) $CD11b^{+}Ly6G^{+}$ cells (neutrophils), (B) $CD11b^{+}CD45^{hi}$ cells (macrophages), $CD11b^{+}CD45^{low}$ (microglia)and (C) macrophage subtype.

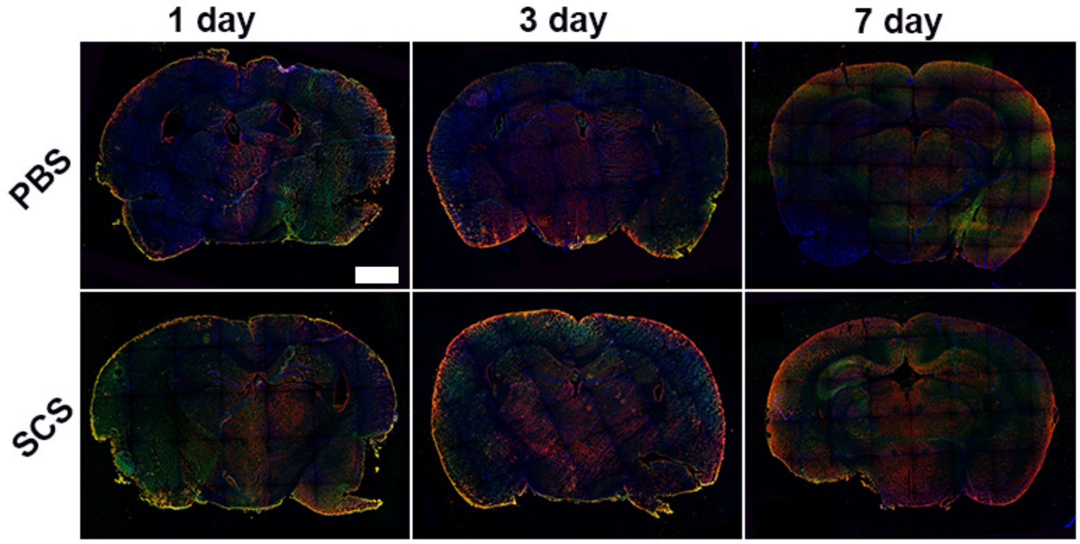

Figure S5. The immunostained images of F4/80 (green) and GLUT (red). Scale bar is 1.5 mm.

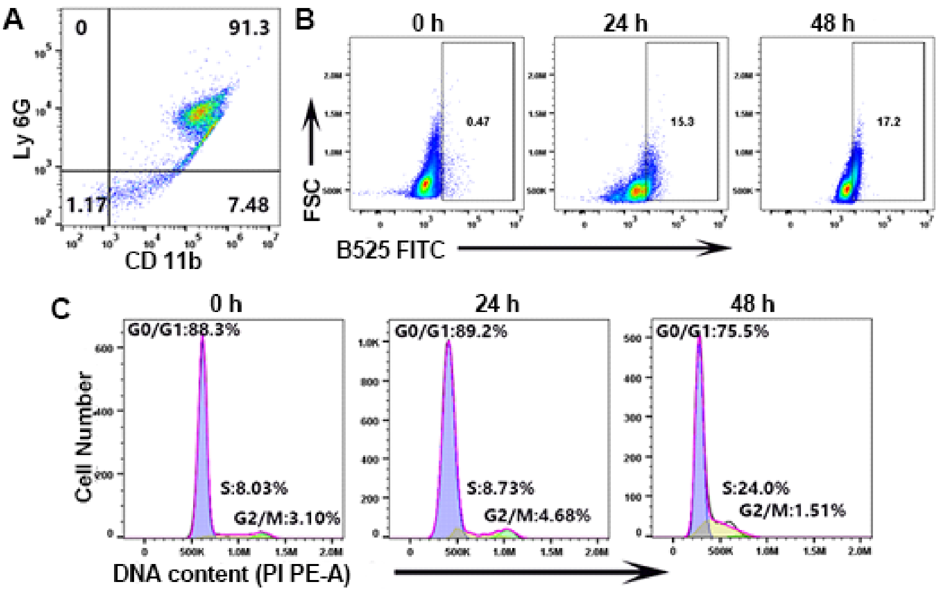

Figure S6. Isolation and activity of neutrophils

(A) Neutrophils ($CD11b^{+}Ly6G^{+}$) were identified by flow cytometry. (B-C) Flow cytometric analysis of neutrophils at different times, (B) SA-β-gal and (C) cell cycle.

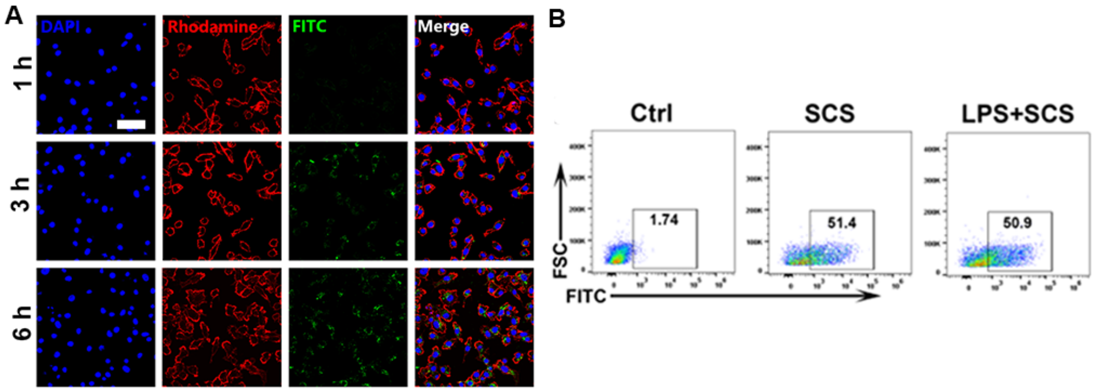

Figure S7. SCS directs the immunomodulation of neutrophils in vitro.

(A) Immunofluorescence and (B) flow cytometry analysis of the binding capacity of SCS on neutrophils.

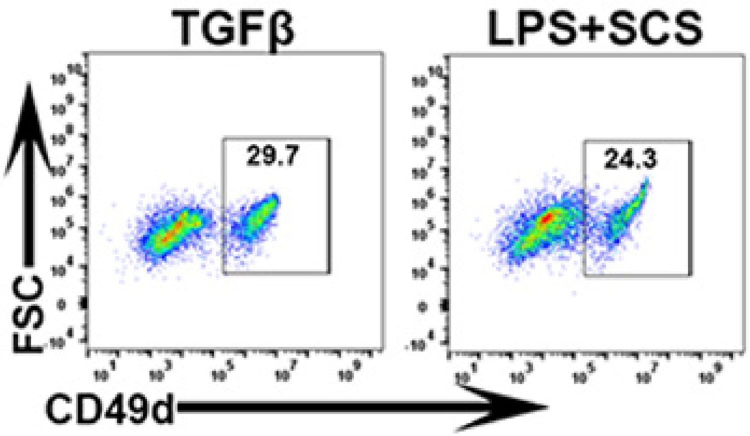

Figure S8. Representative flow cytometry plots of CD49d$^+$ vessel-related neutrophil subsets.

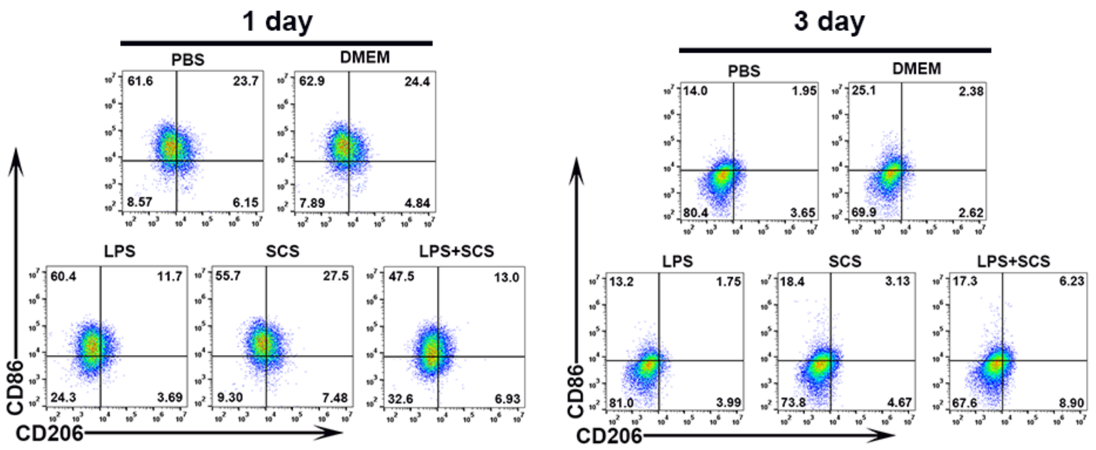

Figure S9. Representative flow cytometry plots of macrophages.

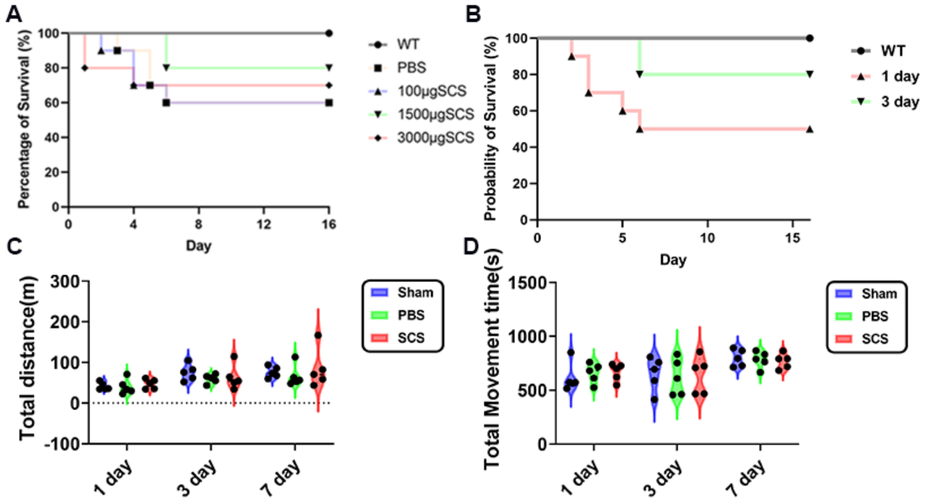

Figure S10. The efficacy of SCS in the treatment of ischemic stroke.

(A) Mortality of mice after treatment with different concentrations. (B) Mortality of mice after treatment with different times. (C) Total movement distance and (D) movement time during the 15 min open field.

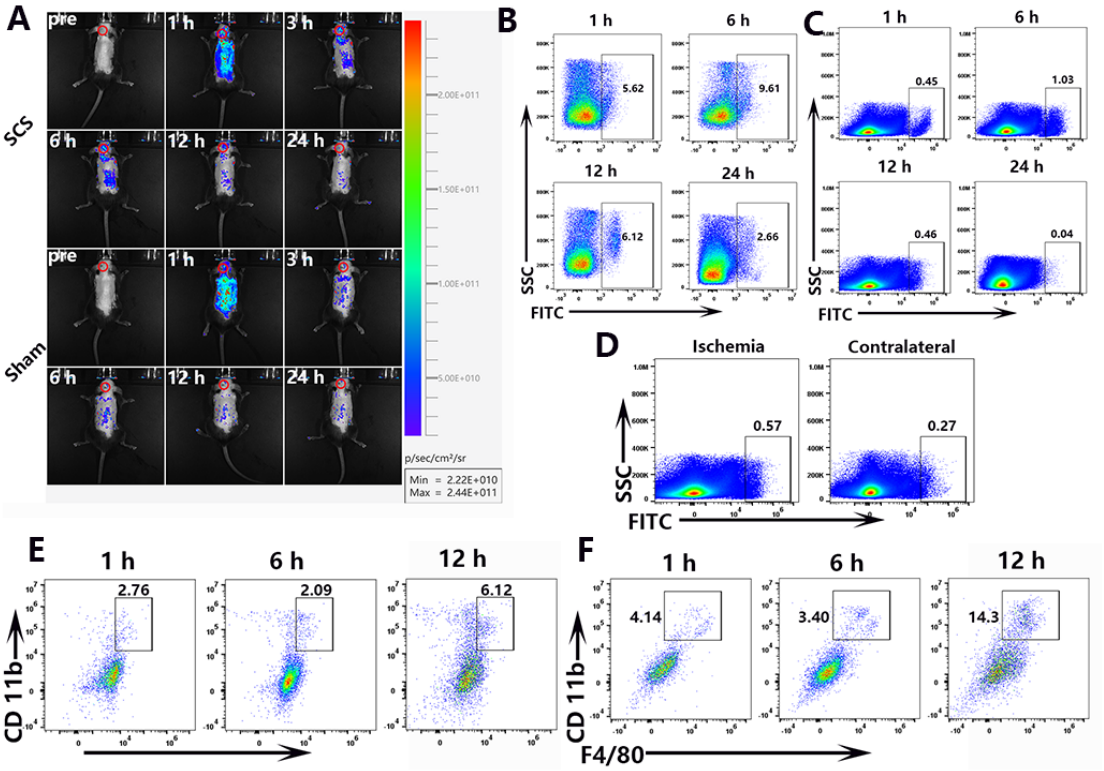

Figure S11. Tracing of SCS after ischemia reperfusion.

(A) Real-time fluorescence imaging of treatment with SCS at different times. (B-C). SCS were traced by flow cytometry in blood (B) and brain tissue (C) at different times. (D) SCS distribution in different brain regions 12 hours after injection. (E-F) Distribution of SCS in neutrophil and macrophage.

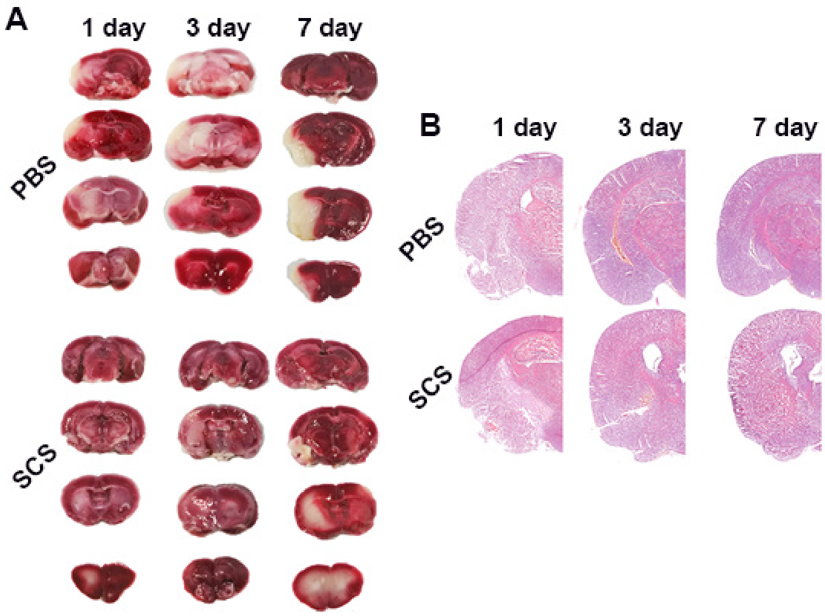

Figure S12. The efficacy of SCS in the treatment of ischemic stroke.

(A) TTC stained coronal brain sections were used for infarction analyses. (B) H&E staining of coronal infarcted brain sections.

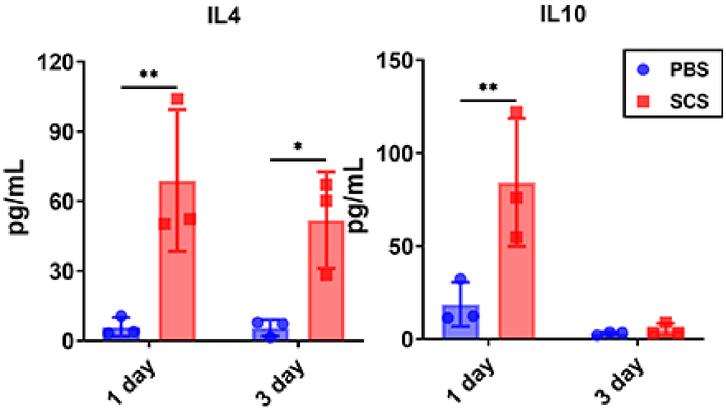

Figure S13. ELISA analysis of IL-4 and IL-10 in blood. Data are shown as mean ± SD (n = 3).

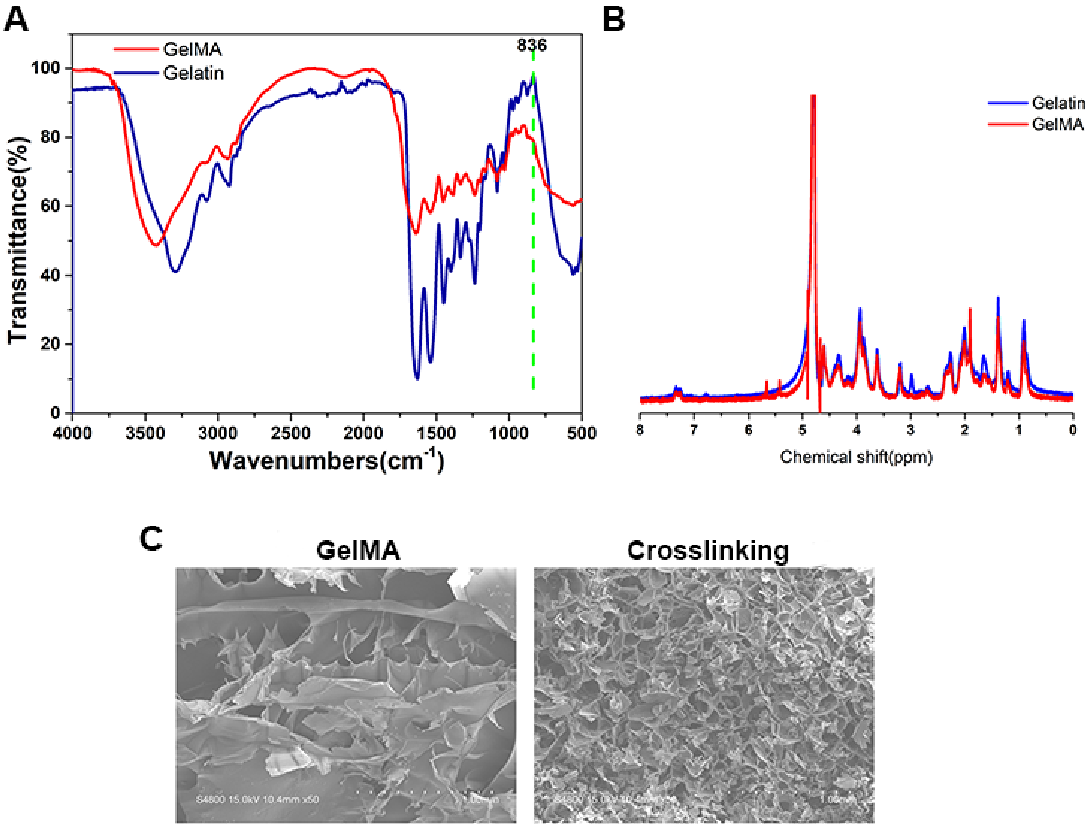

Figure S14. Characterization of GelMA

(A) Fourier Transform Infrared (FTIR) of the gelatin and GelMA. (B) ^{1}H-NMR of the gelatin and GelMA. (C) Scanning electron microscopy (SEM) of GelMA hydrogel.

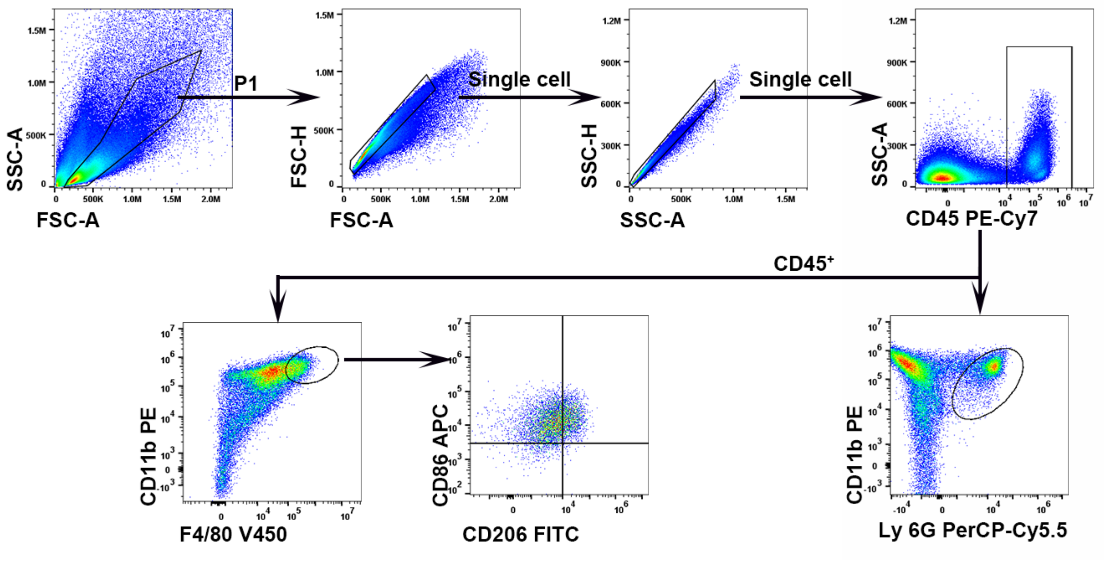

Figure S15. Strategy of sorting neutrophils and macrophages in mouse ischemic hind limb.

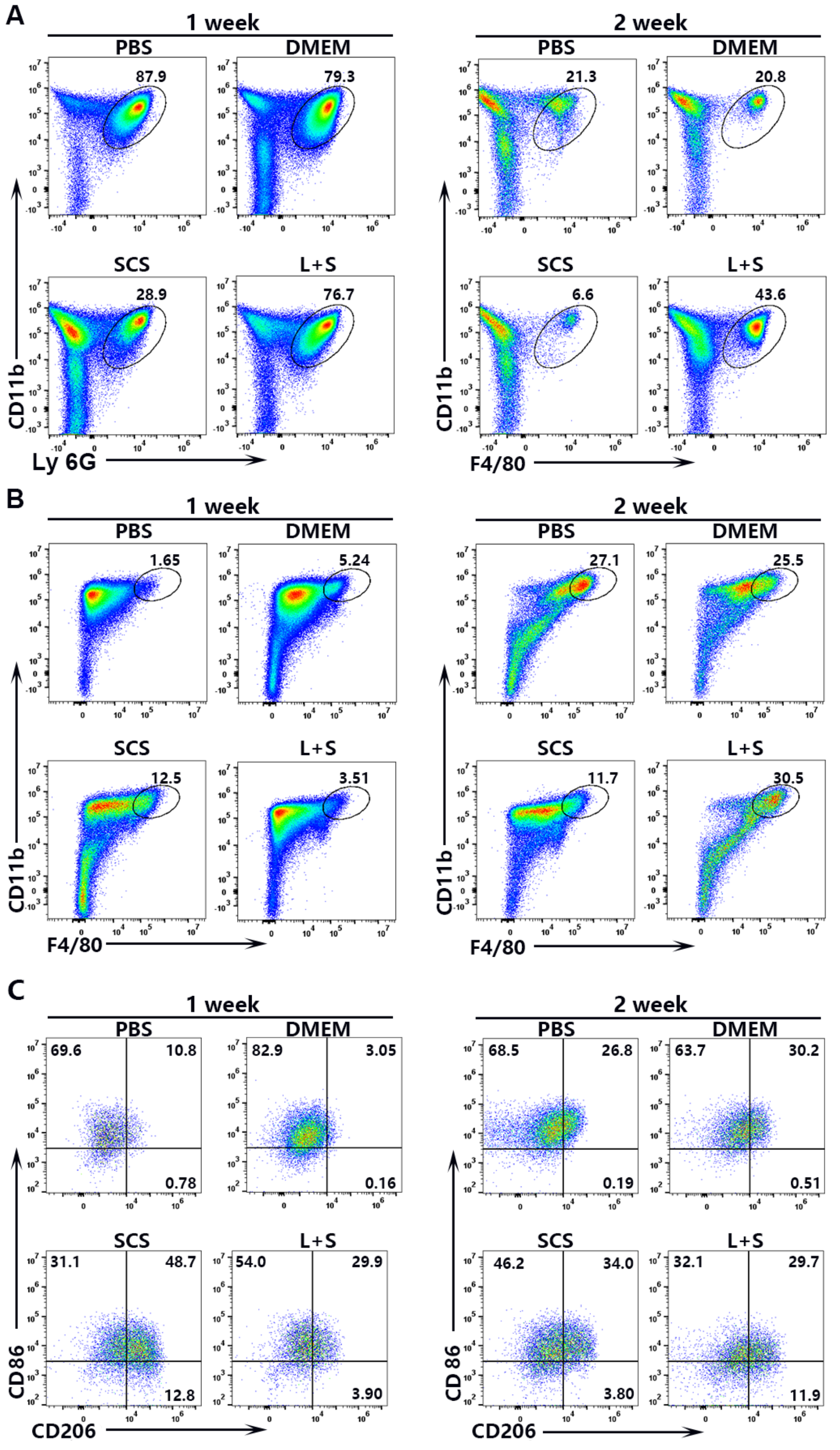

Figure S16. Representative flow cytometry plots in mouse ischemic hind limb.

(A-C) Representative flow cytometry plots of (A) CD11b$^+$Ly 6G$^+$ cells (neutrophils), (B) CD11b$^+$F4/80$^+$ cells (macrophages) and (C) macrophage subtype.

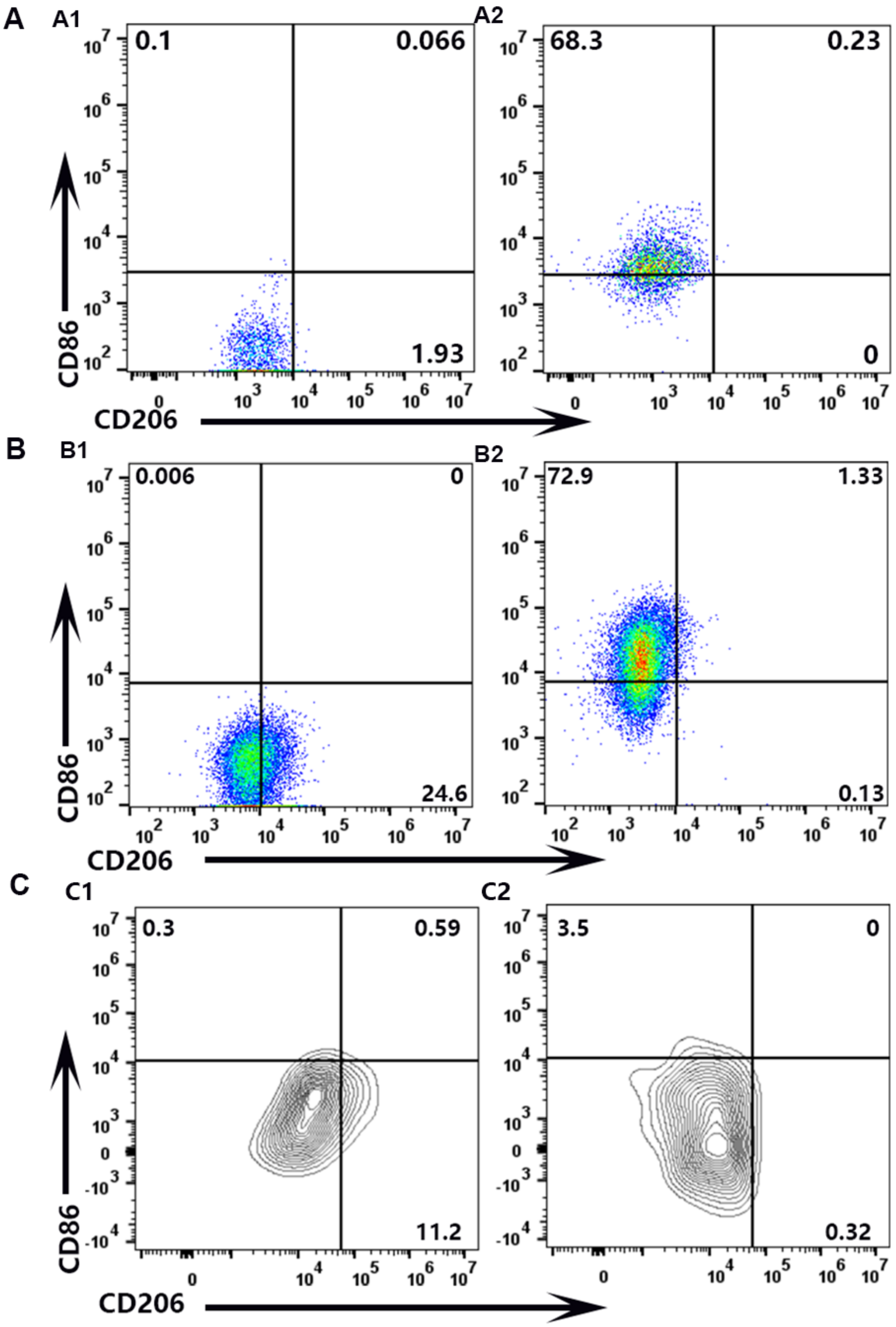

Figure S17. The FMO control of CD86 and CD206.

(A) The FMO control of CD86 (A1) and CD206 (A2) of macrophages in stroke. (B) The FMO control of CD86 (B1) and CD206 (B2) of macrophages in vitro. (C) The FMO control of CD86 (C1) and CD206 (C2) of macrophages in mouse ischemic hind limb.

Supplementary Table S1. Table of genes examined and their primer sequences.

Gene	Direction	Sequence (5’-3’)
Tnf-α	Forward	CAGGCGGTGCCTATGTCTC
	Reverse	CGATCACCCCGAAGTTCAGTAG
Il1β	Forward	TCCAGGATGAGGACATGAGCAC
	Reverse	GAACGTCACACACCAGCAGGTTA
Il6	Forward	ATAGTCCTTCCTAGCCCAATTTCC
	Reverse	GATGAATTGGATGGTCTTGGTCC
Il4	Forward	AGATGGATGTGCCAAACGTCCTCA
	Reverse	AATATGCGAAGCACCTTGGAAGCC
Il10	Forward	GAGAAGCATGGCCCAGAAATC
	Reverse	GAGAAATCGATGACAGCGCC
Tgfβ	Forward	CAGTACAGCAAGGTCCTTGC
	Reverse	ACGTAGTAGACGATGGGCAG
Vegf	Forward	CACGGAGGCAGAGAAAAGAG
	Reverse	CACGGAGGCAGAGAAAAGAG
Pdfbb	Forward	GGAGCCTTGCGGAGGACTGT
	Reverse	GATCTGGGTGCCATCAGAGT
Notch	Forward	AGTGTCAGAGGCCAGCAAGAAGA
	Reverse	TGATTGTCGTCCATCAGAGCACCA
Mmp-9	Forward	GACGTGGGTCGATTCCAAA
	Reverse	GGCAAGTCTTCAGAGTAGTTT
Timp-3	Forward	CGCTCGGCAACTTTGAAGAA
	Reverse	CGGATCACGATGTCGGAGTT
Caspase3	Forward	ATCATTCAGGCCTGCCGGGG
	Reverse	CACGGGATCTGTTTCTTTGCG
Bax	Forward	TTCCGGGTGGCAGCTGACATGTT
	Reverse	TGCTAGCAAAGTAGAAGAGGGCAA
Bcl2	Forward	CATGCCAACGGGGAAACACCAGAA
	Reverse	GTGCTTTGCATTCTTGGATGAAGG
Ccl2	Forward	CAGCACCTTTGAATGTGAACTTG
	Reverse	TGCTTGAGGTGGTTGTGGAA
Ccl3	Forward	TTCTCTGTACCATGACACTCTGC
	Reverse	CGTGGAATCTTCCGGCTGTAG
Ccr2	Forward	TGCTACTCAGGAATTCTCCACAC
	Reverse	GGCCTGGTCTAAGTGCTTGTCAAT
Gapdh	Forward	CGGAGTCAACGGATTTGGTCGTAT
	Reverse	AGCCTTCTCCATGGTGGTGAAGAC

Supplementary Table S2. Antibodies used in the study.

**Antibody**	**Vender**	**Catalogue**	**Dilution***
F4/80	Abcam	Ab6640	1:100 (IF)
CD31	Abcam	ab28364	1:100 (IF)
Ly 6G	Abcam	Ab25377	1:100 (IF)
H3Cit	Abcam	ab219406	1:100 (IF)
GLUT	Abcam	Ab115730	1:100 (IF)
Alexa Fluor 647-conjugated goat anti-mouse IgG	Jackson Immuno.	115-605-00	1:400 (IF)
Alexa Fluor 488-conjugated goat anti-rat IgG	Abcam	150165	1:400 (IF)
Alexa Fluor 594-conjugated goat anti-rabbit IgG	Abcam	150080	1:400 (IF)
Prolong Gold Ant-ifade Reagent with DAPI	Cell Signaling Technology	70658	
LIVE/DEAD™	Thermo	L34976	0.2 μl (FC)
PE-conjugated CD11b	BioLegend	101208	1:100 (FC)
PE/Cy7-conjugated CD45	BioLegend	103114	1:100 (FC)
PerCP/Cy5.5-conjugated Ly6G	BioLegend	127616	1:100 (FC)
BV421-conjugated F4/80	BioLegend	123131	1:100 (FC)
APC-conjugated CD86	BioLegend	105012	1:100 (FC)
FITC-conjugated CD206	BioLegend	141704	1:80 (FC)
GAPDH	Cell Signaling Technology	5174	1:1000 (WB)
Stat3	Cell Signaling Technology	4909	1:1000 (WB)
Phospho-Stat3	Cell Signaling Technology	9145	1:1000 (WB)
NF-κB	Cell Signaling Technology	8242	1:1000 (WB)
Phospho- NF-κB	Cell Signaling Technology	3033	1:1000 (WB)
MyD88	Abcam	ab218413	1:2000 (WB)
MMP-9	Abcam	ab228402	1:2000 (WB)
Anti-rabbit IgG, HRP-linked Antibody	Cell Signaling Technology	7047	1:2000(WB)

*IF: immunofluorescence staining; FC: flow cytometry; WB: western blot
